# Interfacial Thermal Transport of Gold Surfaces Coated with Heterogeneous Monolayers in a Binary Solvent

**DOI:** 10.1021/acsami.5c12225

**Published:** 2025-09-20

**Authors:** Angad Deshmukh, Chao Zeng, James D. E. T. Wilton-Ely, Fernando Bresme

**Affiliations:** † Department of Chemistry, Molecular Sciences Research Hub Imperial College, London W12 0BZ, U.K.; ‡ Thomas Young Centre for Theory and Simulation of Materials, Imperial College London, London SW7 2AZ, U.K.

**Keywords:** thermoplasmonics, molecular simulations, nanoscale heat transport, catalysis, thermodiffusion, interfacial thermal conductance

## Abstract

Gold nanoparticles play a key role in thermoplasmonics due to their efficient light-to-heat conversion and their potential for chemical group functionalization. In this study, we investigate the interfacial thermal transport properties of gold surfaces coated with heterogeneous layers in contact with ethanol–water cosolvents commonly used in catalysis. By employing nonequilibrium molecular dynamics simulations, we quantify the thermal transport characteristics of water–ethanol mixtures at relevant experimental concentrations. Our simulations show excellent agreement with experimental Soret coefficients and reveal that ethanol tends to accumulate in the hot regions at experimentally relevant ethanol–water weight fractions. However, the composition of the solvent layers in contact with the hot gold surfaces is primarily influenced by the interfacial interactions between the substrate and solvent. We examine the interfacial thermal conductance of gold surfaces coated with self-assembled monolayers, including hexanethiol (hydrophobic), mercaptohexanol (hydrophilic), and catalytic units designed to offer an immobilized form of PdCl_2_-(diphosphine). Our findings indicate that the preferential adsorption of ethanol (on hydrophobic surfaces) or water (on hydrophilic surfaces) significantly alters the interfacial thermal conductance. These results help explain recent observations in plasmonic sensing experiments. Furthermore, we demonstrate that in heterogeneous surfaces incorporating hydrophobic spacers and catalytic units, enhanced heat transport occurs, leading to significant temperature differences among the catalytic units, spacers, and the surrounding solvent at nanometer length scales. These insights improve our understanding of thermal and mass transport at catalytic surfaces and will inform the extensive research on thermoplasmonic applications of gold nanomaterials.

## Graphical abstract

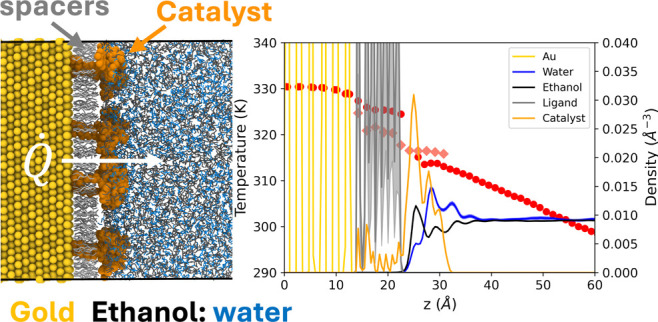

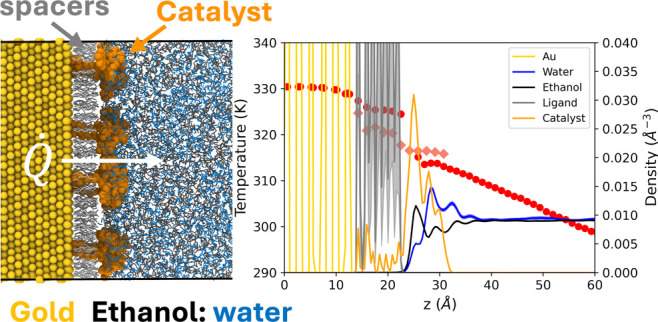

## Introduction

Gold nanomaterials (AuNMs) possess a unique capability to generate intense heat at the nanoscale, leading to the formation of hotspots where local temperatures can often exceed many tens of Kelvin.^1,2^ Furthermore, AuNMs can be functionalized with a wide range of chemical groups, enabling innovative methods for controlling heat transport at subnanometer length scales.^3,4^ The efficient conversion of light into heat in AuNMs can be harnessed in the emerging field of thermoplasmonics,^5^ which holds great promise for advancements in medical, chemical, and imaging applications. Indeed, the biomedical applications of thermoplasmonics have witnessed significant advances, particularly with the introduction of AuNM-based plasmonic photothermal therapies.^6–9^ In contrast, the application of thermoplasmonic technology in other fields, such as catalysis, is still in development.^5^

The use of nanomaterials in catalysis has focused on the inherent catalytic properties of the metal surface, the high surface-to-volume ratio and quantum confinement effects.^10^ The possibility of functionalizing nanomaterials with catalytically active units, e.g. palladium (Pd) ligands, has opened a route to perform carbon–carbon coupling and other reactions, potentially even sequentially. A variety of linkers have been developed with donor groups designed to coordinate catalyst metal units. The majority of approaches employ polydentate donors in order to prevent loss of the metal unit, allowing recycling, which has led to examples bearing diamine, diphosphine or hybrid phosphine-imine chelates, while the strong bonds formed between the metal and N-heterocyclic carbenes have also led to the use of this versatile ligand class.^11–14^

Incorporating thermoplasmonics in catalytic applications requires a good understanding of heat flow through nanoscale surfaces and their interfacial thermal resistance,^15,16^ since the latter influences heat transport across metallic surfaces.^17^ Moreover, the solvents employed in catalytic applications often consist of binary mixtures, such as ethanol–water.^18–20^ Solvent mixtures can significantly influence the outcome of catalytic reactions, offering opportunities to tune activity, selectivity, and reaction rates by modulating solvent–catalyst interactions, solvent–reactant interactions, solubility of different substrates in a single phase and solvent participation in the catalytic cycle.^21^

Despite recent advances, comprehensive studies of thermal transport at functionalized surfaces in contact with water–ethanol cosolvents remain limited. Only recently have experimental data on the interfacial thermal conductance of functionalized gold surfaces in contact with water–ethanol mixtures been reported.^22^ These experiments demonstrated a strong dependence of the interfacial thermal conductance on the ethanol content. However, the microscopic interfacial structure, the relative adsorption of ethanol and water at the surface, and the interfacial temperature profiles have yet to be investigated to achieve a microscopic understanding of the thermal transport in these complex interfaces.

Moreover, local thermal gradients emerging from the heating of AuNMs can induce thermodiffusion, i.e., the Soret effect in multicomponent solvents,^23,24^ which can potentially modify the local composition of the solvent around hot spots in the AuNMs. Previous experiments demonstrated a complex thermodiffusion response in ethanol–water mixtures,^24–26^ highlighting a sign change at high water mass fraction where the Soret coefficient becomes negative, and water accumulation in the hot region. Such sign changes in the Soret coefficient have been confirmed in nonequilibrium molecular dynamics (NEMD) simulations.^27^

Here, we use NEMD simulations^3,28–37^ to investigate the interfacial thermal transport properties of mixed assembled monolayers immersed in water with ethanol as a cosolvent. The ethanol–water mixture is used in catalysis,^38,39^ and has emerged as a green alternative to traditional organic solvents (toluene, cyclohexane, benzene, THF, DMF).^21,40^ Ethanol is readily bioderived through fermentation and, in combination with water, can dissolve a wide range of organic substrates, also imparting advantages in reactivity and selectivity.^21,41^ For example, ethanol–water mixtures are increasingly used as environmentally friendly solvents in catalytic hydrogenation and organic cross-coupling reactions, including the Suzuki–Miyaura reaction.^18,21,40,42^ We report in our work (a) interfacial thermal resistance of AuNMs surfaces coated with monolayers in contact with ethanol–water mixtures, (b) temperature distributions in ligands with complex geometries that mimic those used in metal-mediated catalysis, focusing on heat rates typically employed in experimental settings; and (c) discuss the impact of nanoscale heating on the structure and local composition of the ethanol–water mixture in contact with both bare and functionalized gold surfaces, which are commonly used in catalytic applications.

## Methods

### Theoretical Background

Solid–liquid interfaces exhibit a thermal resistance due to the differing thermal transport properties of the two phases in contact. This thermal resistance is known as thermal boundary resistance or Kapitza resistance, denoted as *R*_K_. The reciprocal of Kapitza resistance is referred to as interfacial thermal conductance (ITC), represented by *G*_K_.

The Kapitza resistance, is related to the “temperature jump”, Δ*T*, across the interface

1 $$R_{K}\equiv \frac{1}{G_{K}}=\frac{A\Delta T}{\mathrm{Q̇}}$$

where *A* is the interfacial area and Q̇ is the heat rate flow perpendicular to the interface.^15,43^

Applying the thermal gradient to a binary mixture induces thermodiffusion, resulting in a mass flux driven by the thermal gradient.^23^ In this work, we investigate the thermal diffusion of ethanol–water binary mixtures. According to linear nonequilibrium thermodynamics, the heat, **J**_*q*_, and mass, **J**_1_, fluxes are defined as^23^

2 $$J_{q}=-L_{qq}\frac{\nabla T}{T^{2}}-\frac{L_{q1}}{T}\nabla_{T}\left(\mu_{1}-\mu_{2}\right)$$

3 $$J_{1}=-L_{1q}\frac{\nabla T}{T^{2}}-\frac{L_{11}}{T}\nabla_{T}\left(\mu_{1}-\mu_{2}\right)$$

where ∇*T* is the thermal gradient, ∇_T_ represents a derivative calculated at constant temperature, the *L*_ij_’s represent the phenomenological coefficients for the heat (*L*_*qq*_) and mass (*L*_11_) diffusive transport, with *L*_*q*1_ ≡ *L*_1*q*_ are the cross phenomenological coefficients, and μ_i_ is the chemical potential of species *i*.^44^ At the stationary state **J**_1_ = 0, the Soret coefficient is defined by

4 $$S_{T}=-\frac{1}{T}{\left(\frac{\nabla \ln\left(w_{1}/w_{2}\right)}{\nabla \ln T}\right)}_{J_{1}=0}$$

where the term *w*_*i*_ is the weight fraction of species *i*.

### NEMD Simulation of Thermal Transport

We calculated the Soret coefficient by analyzing the simulated temperature and composition profiles using NEMD and applying eq 4. Additionally, we used NEMD to determine the thermal conductivity, λ, of the mixture as a function of its composition, employing Fourier’s law

5 $$J_{q}=-\lambda \nabla T$$

where $J_{q}=\mathrm{Q̇}/2A$ is the heat flux, Q̇ is the heat rate flowing through the area *A* and the factor of 2 accounts for the two heat fluxes generated in the simulations (see Figure 1). NEMD simulations take into account all possible thermal coupling effects, making this method ideal for simulating the thermal transport properties of mixtures. When applied to a binary system, NEMD provides a more efficient alternative compared to equilibrium Green–Kubo computations, which require the calculation of several correlation functions (see for example, ref 45).

**Figure 1:**
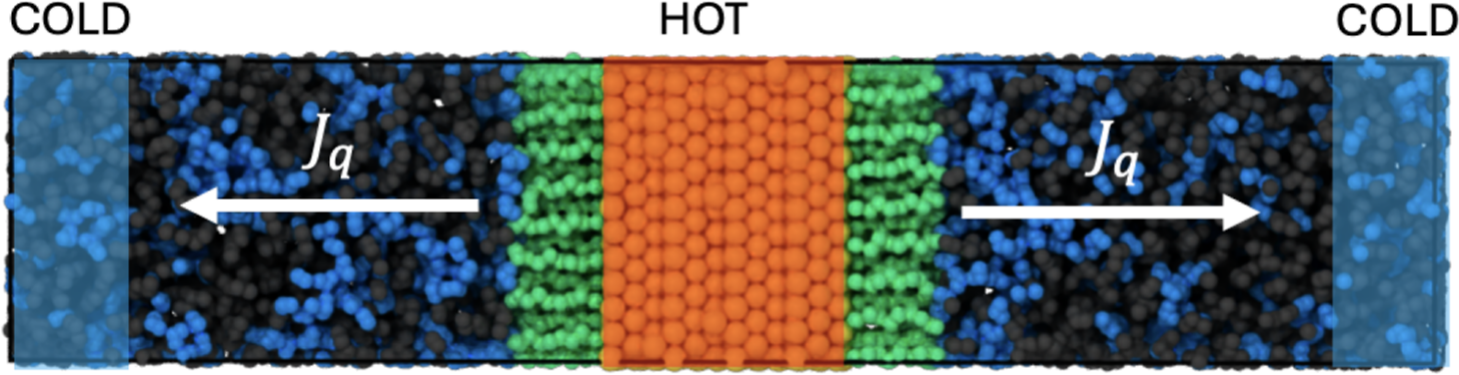
Simulation snapshot of the NEMD simulation showing the setup used to investigate the heat transport across gold-surfactants/fluid interfaces. The snapshot illustrates an example of a mercaptohexanol monolayer (represented by green spheres), adsorbed on a (111) gold surface (the central slab in the image). The surface is immersed in a mixture of ethanol (shown as black spheres) and water (blue spheres) in a 50:50 mol ratio. The blue areas at the edges of the box represent the regions used for the cold thermostat, while the red area in the center of the box indicates the hot thermostat region. The arrows indicate the direction of the heat fluxes (*J*_*q*_) in the simulation cell. The visualizations were generated with Ovito.^46^

To investigate the effects of interfacial thermal transport, we modeled flat slabs of Au(111) immersed in ethanol–water mixtures. We examined pristine and functionalized surfaces as flat slabs to replicate surfaces typically found in large nanoparticles, which usually have diameters in the range of tens of nanometers. The gold surfaces were coated with hydrophobic and hydrophilic alkanethiol ligands to mimic layers with high and low interfacial thermal conductance. Additionally, we explored how these hydrophilic and hydrophobic groups affect the interfacial structure of ethanol–water mixtures. The thermoplasmonic heating is simulated by maintaining a hot thermostat for the entire gold slab and a cold thermostat for the solvent molecules far from the gold surface.^33^ This setup ensures that the system develops composition and temperature profiles that are not influenced by the boundary conditions.

Figure 1 illustrates the setup used for our computations of interfacial thermal conductance. At the center of the simulation box, we placed a gold slab, either in direct contact with ethanol–water mixtures or coated with self-assembled monolayers. To maintain temperature control, we defined thermostat regions at both ends and the center of the simulation box (see Figure 1). The gold atoms were heated to a target temperature, while the ethanol and water molecules entering the cooler regions acted as a heat sink. We employed 350 K (hot) and 300 K (cold) for gold-ethanol/water simulations, and 330 (hot) and 280 K (cold) for the gold surfaces coated with ligands. After several nanoseconds, the system reached a stationary state, resulting in two heat fluxes, $J_{q}=\mathrm{Q̇}/2A$ , flowing in opposite directions. The total simulated heat rate Q̇ was determined from the derivative of the cumulative energy exchanged at the hot and cold thermostats. Additionally, we used simulation boxes filled only with ethanol and water to calculate the thermal conductivity and Soret coefficients of the ethanol–water mixtures.

We coated the gold surfaces with thiolates, specifically hexanethiol (hydrophobic) and mercaptohexanol (hydrophilic). We also included ligands with chemical compositions relevant to catalysis in some simulations. We selected a disulfide-terminated lipoic acid chain, where the disulfide opens to form a dithiolate attachment to the gold surface. The carboxylic acid unit allows conjugation of a wide range of functionality.^47,48^ The headgroup of this ligand consisted of bulky aromatic rings mimicking the structure of catalytic units typically employed in catalysis bearing phosphines and, in particular, diphosphines.^49^ The chemical structure of the ligand is shown in Figure 2. In the presence of this ligand, the alkanethiol molecules act as spacers, which help prevent steric congestion between adjacent catalytic units and inhibit direct contact between the solvent and the gold (Au) surface. We analyzed the temperature profiles along the ligand chains by examining the NEMD trajectories. This analysis enabled us to investigate the temperature distribution in the local environment surrounding the catalytic units attached to the thermoplasmonic material.

**Figure 2:**
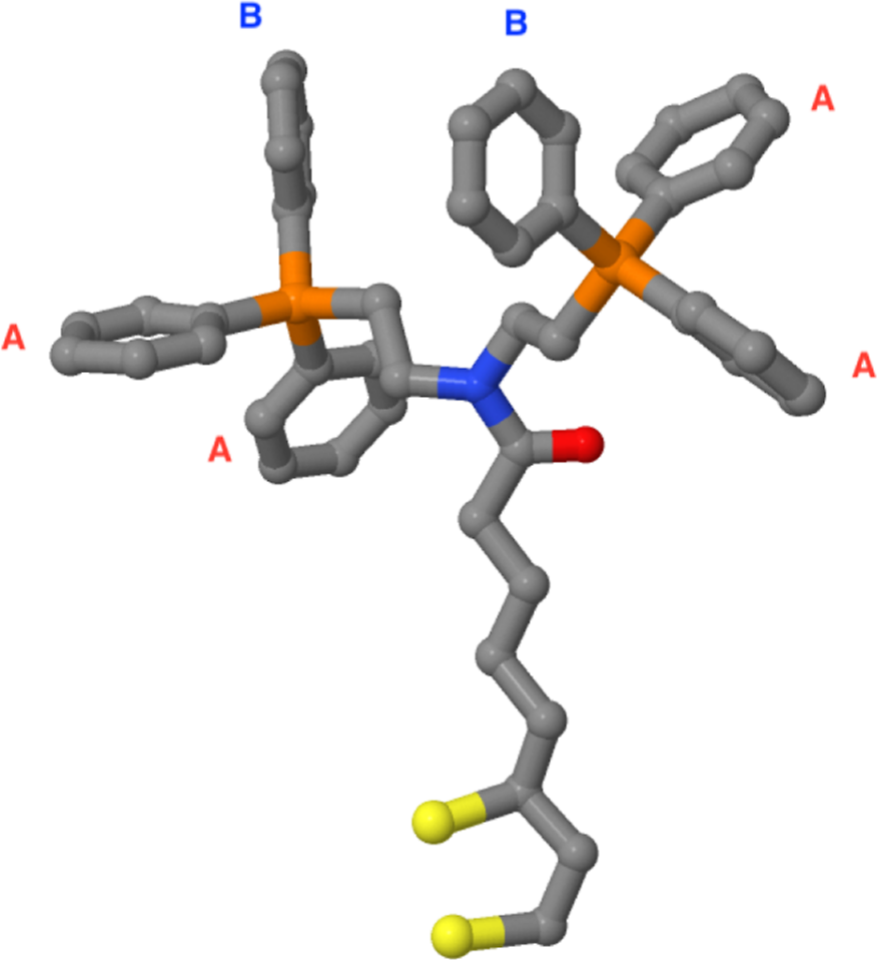
MD ligand structure used to simulate the gold functionalized surfaces. The ligand composition is $S_{2}C_{3}H_{5}{\left(CH_{2}\right)}_{4}C\left(O\right)N{\left\{CH_{2}CH_{2}P{\left(C_{6}H_{5}\right)}_{2}\left(C_{6}H_{5}\right)\right\}}_{2}$ The aromatic rings labeled “A” create steric bulk around the metal center. In a palladium (Pd) catalyst, the Pd center would be positioned in place of the aromatic rings labeled “B”. Phosphorus, nitrogen, oxygen, sulfur, and carbon are represented in orange, blue, red, yellow, and gray, respectively. The hydrogen atoms are not depicted in this molecular structure.

The simulations were conducted using the LAMMPS package,^50^ with the TraPPE^51^ and TIP4*P*/2005 ^52^ force fields to model the ethanol and water molecules, respectively. Ethanol molecules were represented using a fully flexible united-atom model. The gold slab was modeled using a force field developed by Heinz et al.,^53^ while the interactions of the alkanethiol ligands were calculated using the force fields discussed in ref 3. In this model, the –OH terminated ligands are hydrophilic, exhibiting a water contact angle of 40°; conversely, the methyl (–CH_3_) terminated ligands are hydrophobic with a water contact angle of 120°,^3^ values which are in good agreement with experimental studies.^54^ For the simulations involving the catalytic unit, we employed the GROMOS 54A7 force field.^55^

Our simulations were conducted using flat gold surfaces, which is appropriate for nanomaterials with dimensions on the order of tens of nanometers. However, local roughness and curvature may affect interfacial thermal transport and generally lead to an increase in interfacial thermal conductance.^29,56^ This aspect is not addressed in the present work.

The equations of motion were integrated using a time step of 0.5 fs. A typical NEMD simulation lasted 2.5 ns, with five independent repeats resulting in a total simulation time of 12.5 ns per system. The protocol we followed included the following steps: (a) The system was first equilibrated at constant temperature and pressure in the NpT ensemble. (b) Hot and cold thermostats were established in discrete regions of the simulation box to generate the heat fluxes. (c) During the production phase, the simulation box was divided into slabs perpendicular to the direction of the heat flux. These slabs were used to calculate local temperature, density, and composition profiles throughout the simulations. The results from the various repeats were then used to calculate standard errors. Additional details regarding the simulation conditions and protocols can be found in the Supporting Information.

## Results and Discussion

### Thermophysical Properties of Ethanol–Water Mixtures

To assess the accuracy of the force field used for simulating the ethanol–water mixtures, we calculated various thermophysical properties and the Soret coefficient of the mixture at different weight fractions of water and ethanol. These simulations were carried out at 300 K, targeting pressures close to 1 bar. Further details on the simulation conditions are provided in Tables S3 and S4 of the Supporting Information.

Figure 3 shows the density of the mixture as a function of the ethanol mole fraction. The simulation results align well with the experimental data. The two experiments presented in Figure 3 show differences at intermediate ethanol mole fractions. Our findings agree with the data of Pečar and Doleček^57^ and the computer simulation data reported by Benavides Bautista et al.^58^ The density of the mixture decreases as the ethanol composition increases. The nonlinear dependence of the density on mole fraction is consistent with nonideal mixing.^59^

**Figure 3:**
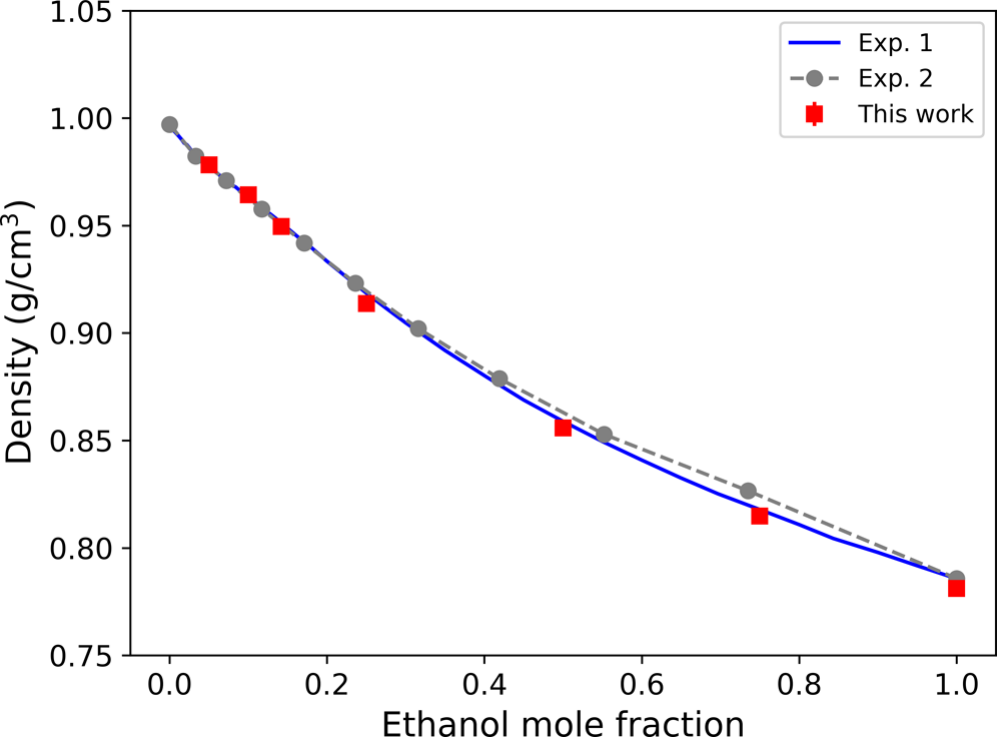
Density of ethanol–water mixtures as a function of the ethanol mole fraction simulated at a pressure of 1 bar and a temperature of 300 K. The error bars on the data are smaller than the size of the symbols used. The corresponding numerical data are reported in Table S3 in the Supporting Information. The experimental data, Exp. 1, are taken from ref 57 and Exp. Two from ref 59.

We computed the thermal conductivity of the ethanol–water mixture as a function of its composition, using NEMD simulations and Fourier’s eq (eq 5). The simulation conditions, including average temperature and pressure, for various ethanol–water mixtures are presented in Table S4 in the Supporting Information.

The experimental thermal conductivity of pure ethanol is significantly lower than that of pure water at standard conditions, with values of 0.171 W/(Km) for ethanol and 0.607 W/(Km) for water.^60^ This disparity leads to a substantial dependence of a solution’s thermal conductivity on its solvent composition, as ethanol exhibits greater resistance to heat transport compared to water.

Figure 4 shows the dependence of thermal conductivity on ethanol mole fraction. The results align well with the experimental findings reported in ref 61 demonstrating a rapid decrease for mole fractions below 0.2, followed by a slower reduction at higher mole fractions. The TIP4P/2005 water model is known to overestimate the thermal conductivity of pure water,^63^ and this overestimation of thermal conductivity is also evident at low mole fractions of ethanol–water mixtures. Regarding thermoplasmonic heating, the reduced thermal conductivity of the ethanol–water mixtures is expected to result in stronger thermal gradients under identical heat flux experimental conditions.

**Figure 4:**
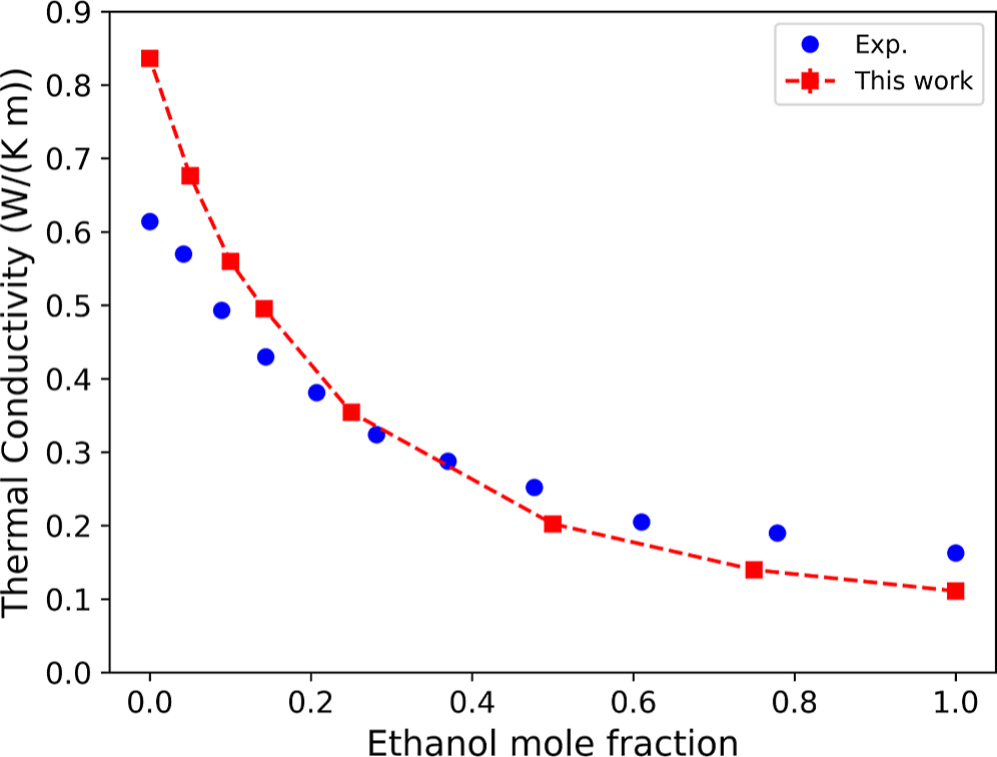
Dependence of thermal conductivity on the composition of ethanol–water mixtures. For numerical data, refer to Table S4 in the Supporting Information. The label “Exp.” denotes the experimental data from previous work.^61^ The simulated thermal conductivity for pure water is taken from a previous study.^62^ The dashed line is a guide to the eye. The simulated thermal conductivity for pure ethanol is 0.111 ± 0.003 W/(K m).

### Thermodiffusion of Ethanol–Water Mixtures

In this section, we discuss the thermal diffusion of ethanol–water mixtures. The NEMD computations involve applying a thermal gradient to a nonequilibrium system that remains in a steady state with zero mass flux. By calculating the local composition and the local thermal gradient, we can determine the Soret coefficient using eq 4, which can be reformulated in terms of mole fractions:

6 $$S_{T}=-\frac{1}{x_{1}x_{2}}{\left(\frac{dx_{1}}{dT}\right)}_{J_{1}=0}$$

where *x*_*i*_ represents the mole fraction of component *i*, and the calculation involves a local derivative of the composition with respect to temperature.

We analyzed the relationship between composition and temperature in the direction of the heat flux (see Figure 5). We calculated the Soret coefficient using eq 6, using the local composition gradients at the temperatures of interest. Due to the slow rate of the diffusive flux, the simulations required between 50 and 100 ns to reach the steady-state condition. We averaged the composition and temperature profiles obtained from both sides of the simulations, corresponding to the two opposing heat fluxes.

**Figure 5:**
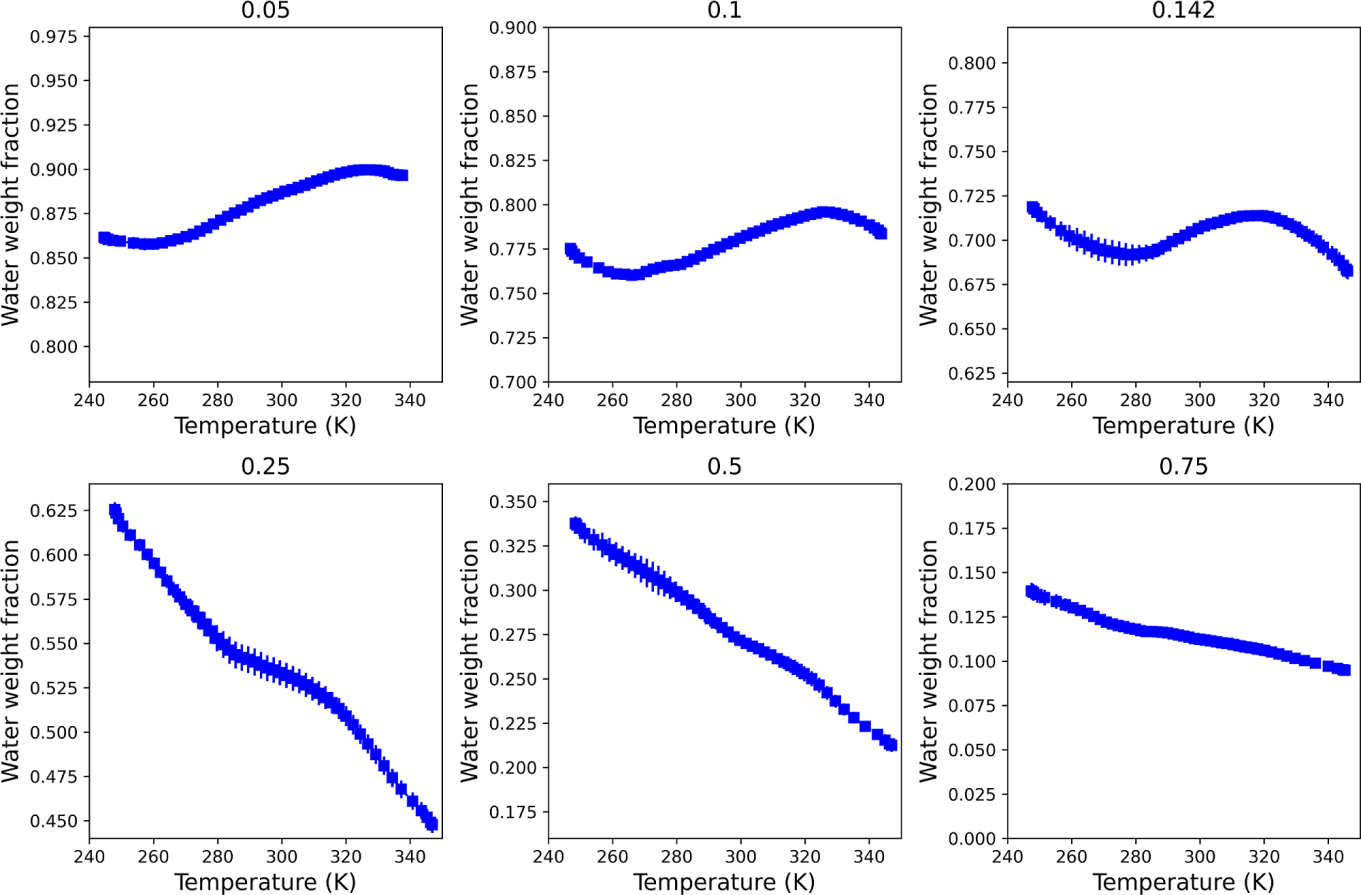
Profiles of water weight fractions from NEMD simulations of ethanol–water mixtures at various compositions. Each panel is labeled with the average ethanol mole fraction, as follows: 0.05 (0.881), 0.1 (0.778), 0.142 (0.702), 0.25 (0.538), 0.5 (0.28), 0.75 (0.115), where the number in brackets represents the corresponding water weight fraction, $w_{H_{2}O}$ . The error bars represent the standard error derived from three independent simulations.

The systems with lower ethanol mass fraction reached the steady-state conditions more rapidly. The simulation times to define the steady-state were as follows: 45 ns for mixtures with ethanol mole fractions of 0.05, 0.1, and 0.142, compared to 105 ns for systems with ethanol mole fractions of 0.25, 0.5, and 0.75. These differences in time correspond well with the diffusion coefficients of water, which are approximately 2.5 times higher than those of ethanol, based on the force fields used in this study (see previous work^58,64^ ).

We show in Figure 5 the dependence of the water mass fraction on temperature. The plots represent pairs of local weight fractions and temperatures. A positive slope indicates that mass transport occurs against the direction of the thermal gradient, indicating that the concentration of water increases in the hotter region. Mixtures with a high water composition, specifically a weight fraction of $w_{H_{2}O}\geq 0.778$ , are classified as thermophilic, and their Soret coefficient is negative. In these cases, water is preferentially transported toward the hotter region. In contrast, at lower water weight fractions $w_{H_{2}O}\geq 0.538$ , the Soret coefficient becomes positive, leading to the accumulation of water in the cold region; this indicates that the solution exhibits thermophobic behavior toward water.

Experimental measurements of the Soret coefficient, conducted using forced Rayleigh scattering and beam deflection cells,^24,25^ indicate that the Soret coefficient is approximately zero for water mass fractions between $w_{H_{2}O}=0.65$ and 0.7. In this range, the behavior of water in the mixtures shifts from thermophobic to thermophilic. Our computations also reveal a crossing point in the Soret coefficient. This is illustrated in the panels for compositions ranging from 0.1 to 0.142 and from 0.25 to 0.75 in Figure 5, which show a change in slope occurring between $w_{H_{2}O}=0.778$ to 0.538, in agreement with experimental results.

The data presented in Figure 5 also show that the strength of the Soret effect, indicated by the slope of the weight fraction-temperature lines, varies significantly with the composition of the mixture. Notably, the slope change is more pronounced for the average compositions of $w_{H_{2}O}=0.538$ and 0.28, suggesting a stronger coupling between thermal and mass transport in these mixtures.

We calculated the Soret coefficients in the temperature range of 280 to 320 K by using the local gradient $dw_{H_{2}O}/dT$ and the compositions presented in Figure 5. In Figure 6, we compare our Soret coefficients with previous experimental data obtained at 293 K. It is important to note that the variation in the experimental data between 293 and 298 K is small.^24^ Our results are in good agreement with the experimental findings reported in previous works.^24,25^

**Figure 6:**
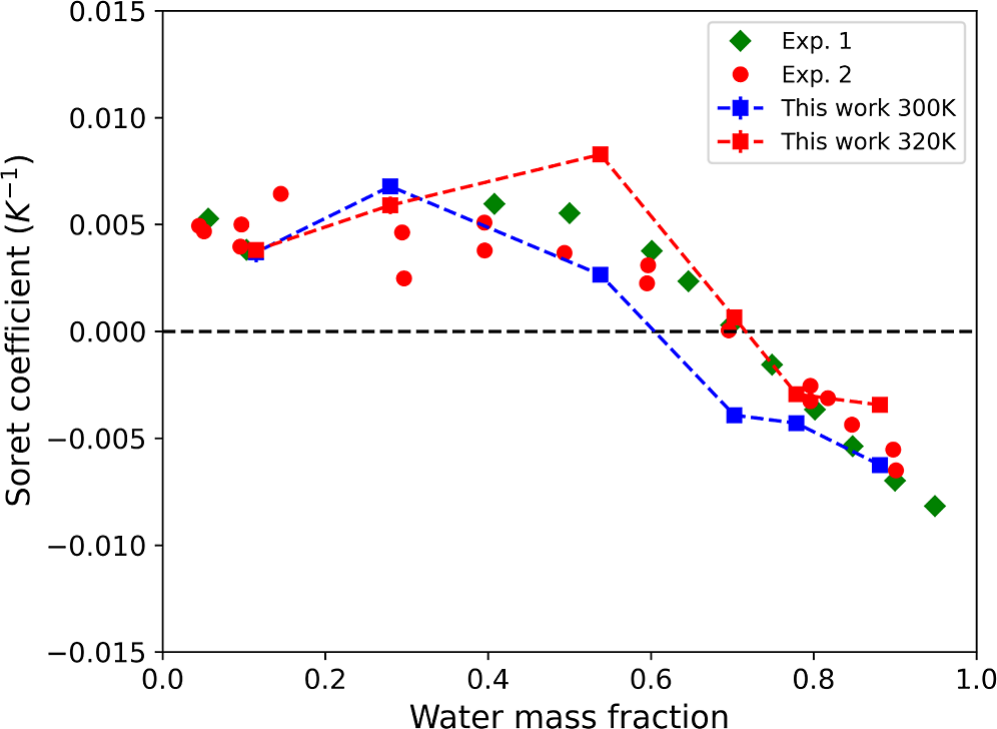
Soret coefficient of the ethanol–water mixtures. The squares represent the NEMD data obtained at temperatures of 300 and 320 K. For further details on the thermodynamic conditions, refer to Table S4. The numerical data for the simulated Soret coefficients can be found in Table S5. “Exp. 1” correspond to data reported in ref 25 at 293.2 K, and “Exp. 2” to data reported in ref 24 at 295.2 K.

We therefore conclude that the force fields used in this study reproduce the general thermodiffusion behavior of water–ethanol mixtures. Specifically, the thermophobicity toward water at low and medium water mass fractions and thermophilicity at high water mass fractions. As the water mass fraction increases, thermodiffusion decreases, eventually becoming zero at $w_{H_{2}O}∼0.6$ . At this point, the mixture transitions from a thermophobic to a thermophilic water response.

We also performed additional calculations for a higher temperature isotherm of 320 K. In this case, the thermophobic/thermophilic transition is observed at $w_{H_{2}O}∼0.7$ , which closely aligns with experimental observations (see Figure 6).

### Interfacial Thermal Conductance of Gold–Ethanol/Water Interfaces

We have characterized the thermodiffusion response of ethanol–water mixtures and demonstrated that our models successfully reproduce their overall experimental behavior. We now focus on the interfacial heat transport of the ethanol–water mixture in contact with a gold (111) surface. We simulated a 50:50 mole fraction of ethanol and water, which is of interest in experimental studies of catalysis. The data reported below were obtained using NEMD simulations, with an average pressure normal to the interface close to 1 bar, *P* = 33.4 ± 3.1 atm, and *T* = 318.9 ± 0.1 K.

Figure 7a shows the temperature and density profiles of gold (Au) and the ethanol–water mixture along the direction of the heat flux. A noticeable temperature jump occurs across the Au-solvent interface at the heat rates used in the simulations, ∼10^–2^ μW. Ethanol tends to adsorb onto the gold surface, leading to a depletion of free water molecules at the surface. Figure 7b shows that the first layer of solvent in contact with gold primarily consists of ethanol molecules. Additionally, we observe a slight enrichment of water toward the cooler region, which is consistent with the positive sign of the Soret coefficient (thermophobic toward water, see Figure 6) at a 50:50 mole fraction (equivalent to a 0.281 water weight fraction).

**Figure 7:**
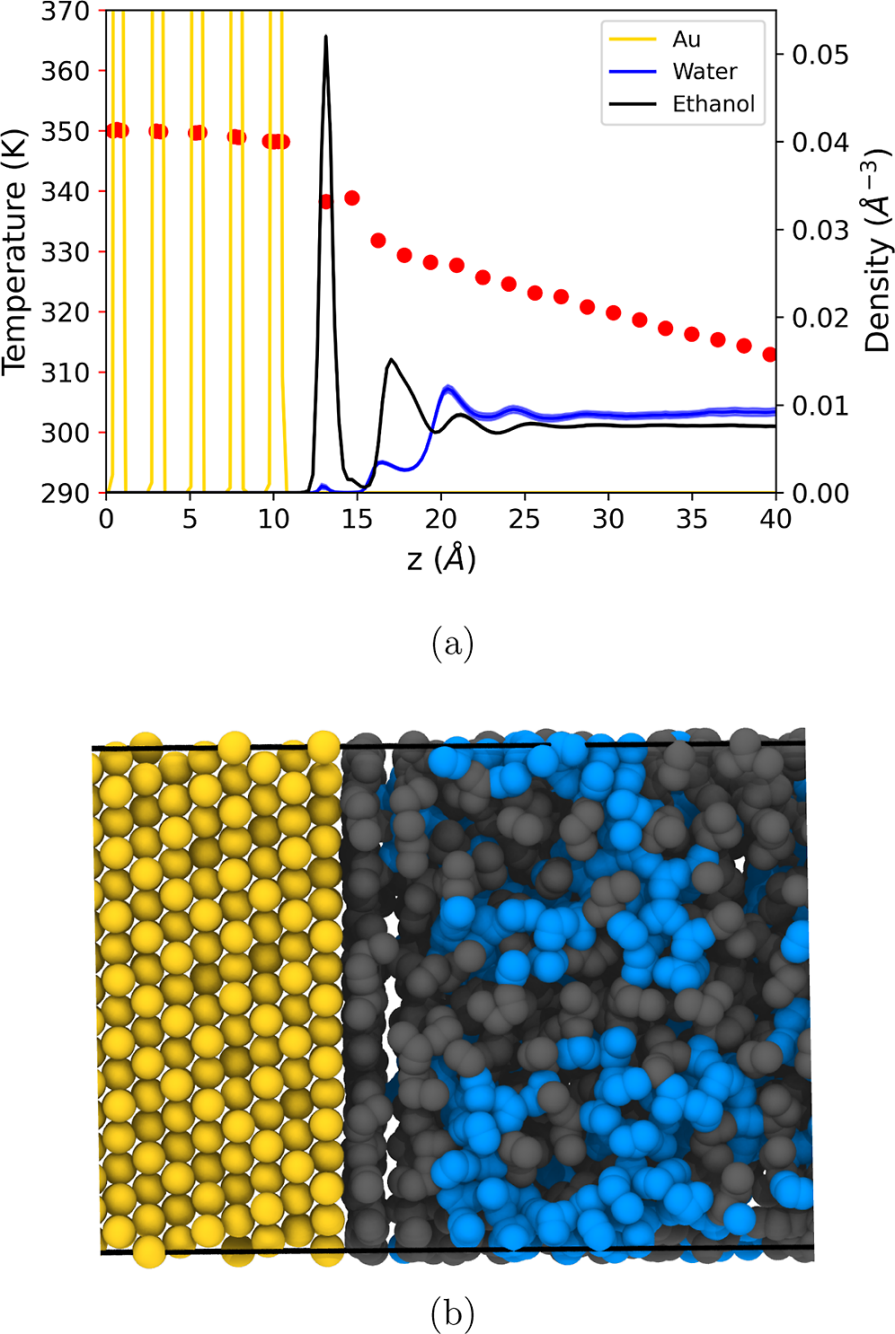
(a) Temperature (left *y*-axis and red circles) and density profiles (right *y*-axis and full llines) for gold and the solvent. The densities are in number of molecules per Å^3^. (b) Snapshot of the interfacial layer of solvent adsorbed on the gold surface, with ethanol and water molecules represented in gray and blue, respectively. All the results correspond to an average composition of 50:50 ethanol–water mole fraction.

We computed the interfacial thermal conductance, *G*_K_, using eq 1, where the heat fluxes *Q̇*/A were typically of the order of ∼10^9^ W/m^2^. The temperature jumps, Δ*T* (see Figure 7a), were determined by fitting the interfacial data points within 0.5 nm of the gold surface. For the solvent, we also included a fitting region of 0.5 nm from the main ethanol peak. Our method closely follows the approach described in previous work.^33^ Linear fitting was used to extrapolate the gold and solvent temperatures to the plane between the gold surface and the first solvent layer; the resulting temperature difference defines Δ*T*.

The interfacial conductance of the water-gold interface has been previously computed using NEMD simulations. Reported values are typically in the range of 200–300 MW/(Km^2^),^32,33^ which are characteristic of hydrophilic interfaces. We conducted additional simulations for pure water-gold and pure ethanol-gold interfaces (refer to Figure S4 in the Supporting Information for temperature and density profiles). The conductance of our water-gold interface was found to be *G*_K_ = 250.9 ± 10.2 MW/(Km^2^), which aligns with values reported in previous studies.^32,33^

In contrast, the conductance of the ethanol–gold interface was lower, *G*_K_ = 104.3 ± 9.5 MW/(Km^2^), thus indicating a greater resistance to interfacial heat transport. Previous studies of ethanol–gold interfaces reported even lower thermal conductances, ∼14 MW/(Km^2^).^65^ The differences between our results and those reported previously may be attributed to the surface–ethanol interactions in that study being adjusted through parametric studies, resulting in ethanol-gold adsorption layers with consistently lower densities than those reported here. Experimental studies suggest that the thermal conductance of the ethanol–gold interface is significantly higher, in the order of 90 MW/(Km^2^), approaching the order of magnitude reported here.^22^

For the 50:50 mole fraction ethanol–water mixture, we obtained a thermal conductance of *G*_K_ = 146.6 ± 7.6 MW/(Km^2^). This value is significantly lower than the conductance observed at the gold–water interface. This reduction in conductance, relative to pure water, agrees with recent plasmonic sensing experiments showing a systematic decrease in conductance with increasing ethanol content.^22^ For ethanol–water mixtures corresponding to our mole fractions, the conductance is ∼90 MW/(K m^2^), indicating a reduction of about 30% compared to the interfacial thermal conductance of pure water measured in the same study. Our simulations show a 40% decrease in thermal conductance for the ethanol–water mixture compared to pure water, which is consistent with the experimental findings.

### Thermal Transport Across Functionalized Interfaces

In this section, we examine heat transport across functionalized gold surfaces. We coated these surfaces with hydrophobic (hexanethiol) and hydrophilic (mercaptohexanol) ligands to analyze how hydrophilicity affects the structure of the ligand–water–ethanol interface and the interfacial thermal transport. We focused our studies on understanding the microscopic thermal transport mechanisms at the interface between functional materials and multicomponent solvents.

The thermal boundary resistance between Au, ligands, and solvent was modeled using a series of thermal circuits.^3,66^ The effective interfacial thermal conductance is given by

7 $$\frac{1}{G_{total}}=\frac{\Delta T_{Au-ligand}}{J_{q}}+\frac{\Delta T_{m}}{J_{q}}+\frac{\Delta T_{ligand-solvent}}{J_{q}}$$

where, Δ*T*_α–β_ are the interfacial temperature-jumps, Δ*T*_m_ is the temperature drop within the monolayer and *J*_*q*_ is the heat flux normal to the functionalized surface.

To generate the thermal gradients, we applied thermostats in the hot and cold regions, *T*_HOT_ = 330 K and *T*_COLD_ = 280 K, respectively. The temperature profiles were fitted using linear functions to calculate temperature jumps in the planes between the different components, gold-ligand, and ligand-solvent. We fitted interfacial temperatures in a region of 0.5 nm from the gold surface, and 0.5 nm from the main solvent peak.

Figure 8a shows the temperature and number density profiles for the hexanethiol-coated (hydrophobic) gold surface. The simulations indicate a stronger tendency for ethanol molecules to adsorb on the hydrophobic monolayer (refer to Figure 8c for an illustration of the interfacial structure). Because the monolayer is fully covered, there is no noticeable penetration of ethanol molecules into this region.

**Figure 8:**
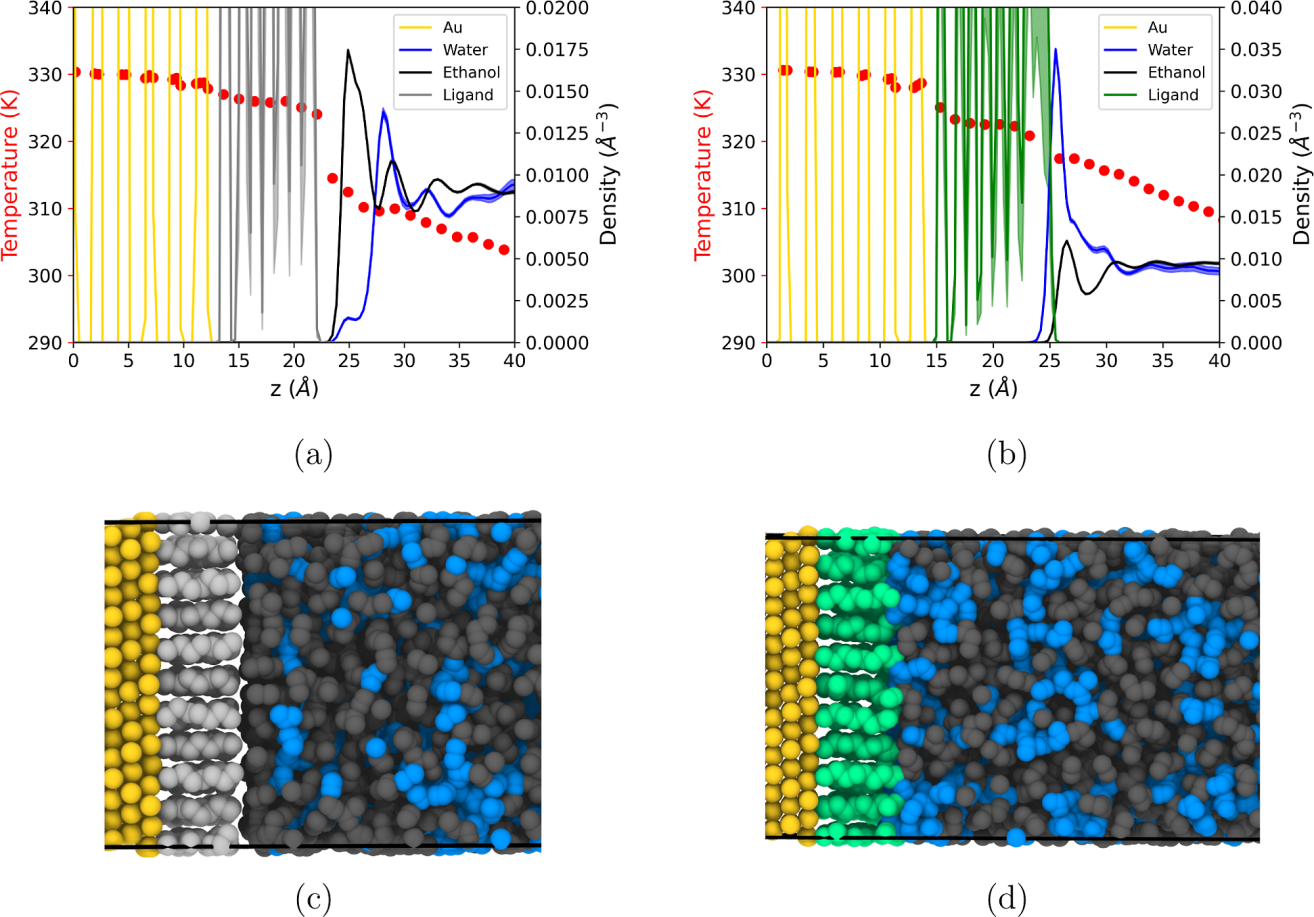
Temperature (red circles) and number density profiles (full lines) for (a) hexanethiol-coated and (b) mercaptohexanol-coated gold surfaces. The error bars indicate standard errors calculated from three replica simulations. Panels (c,d) show simulation snapshots of the hexanethiol and mercaptohexanol systems, respectively. From left to right: gold (yellow), ligand (mercaptohexanol in light gray or hexanethiol in green) and the solvent, which consists of ethanol (dark gray) and water (blue).

The temperature profile shows a significant jump at the ligand–solvent interface, suggesting considerable thermal resistance in that region. Using eq 7, we calculated the total interfacial thermal conductance of the hydrophobic surface, *G*_K_ = 139.3 ± 10.2 MW/(Km^2^). This conductance is dominated by the greater resistance at the monolayer–solvent interface. Both the gold-monolayer and the hexanethiol–monolayer interfaces exhibit high conductivity, with values comparable to those reported in previous NEMD simulations (see Table S6).^3^

The total interfacial thermal conductance of the hydrophobic monolayer is lower than that of gold with the water–ethanol mixtures, but the changes are relatively small. This observation agrees with recent experimental observations using alkanethiolate-coated gold surfaces.^22^ Overall, our results show that the reduction in the thermal conductance observed in experiments is connected to a preferred adsorption of ethanol molecules at the phobic interfaces and water at the philic interface.

The interfacial structure of the hydrophilic monolayers is quite different, dominated by a stronger adsorption of water and a significant depletion of ethanol from the hydrophilic head region of the mercaptohexanol ligand (see Figure 8b,d). In comparison, the solvent layer in contact with the monolayer exhibits a more heterogeneous composition in the hydrophilic layer than in the hydrophobic layer (see Figure 8d), with both components, water and ethanol, in contact with the monolayer, albeit water is the majority component. The OH-terminated hydrophilic ligand forms strong hydrogen bonds with water,^3,31^ enhancing the surface–solvent interactions. These interactions are reflected in the interfacial conductance of the ligand–solvent interface, which is dominant and very high >10^3^ MW/(Km^2^), in agreement with previous studies (see Table S6).^67^

The strong ligand–water interactions are evident in a significantly higher total thermal conductance at the gold-philic ligand–solvent interface than the hydrophobic monolayer. The general behavior observed here aligns with the available experimental data, which also showed a larger change in thermal conductance induced by the incorporation of hydrophilic monolayers.^22^

Figure 9 summarizes our results for the interfacial thermal conductance of the various interfaces investigated in this study. We present data for a bare gold surface in contact with pure water, pure ethanol, and a 50:50 mole fraction mixture of water and ethanol. Additionally, the figure includes conductance measurements for gold surfaces coated with hydrophobic and hydrophilic ligands in contact with the 50:50 mole fraction water–ethanol mixture.

**Figure 9:**
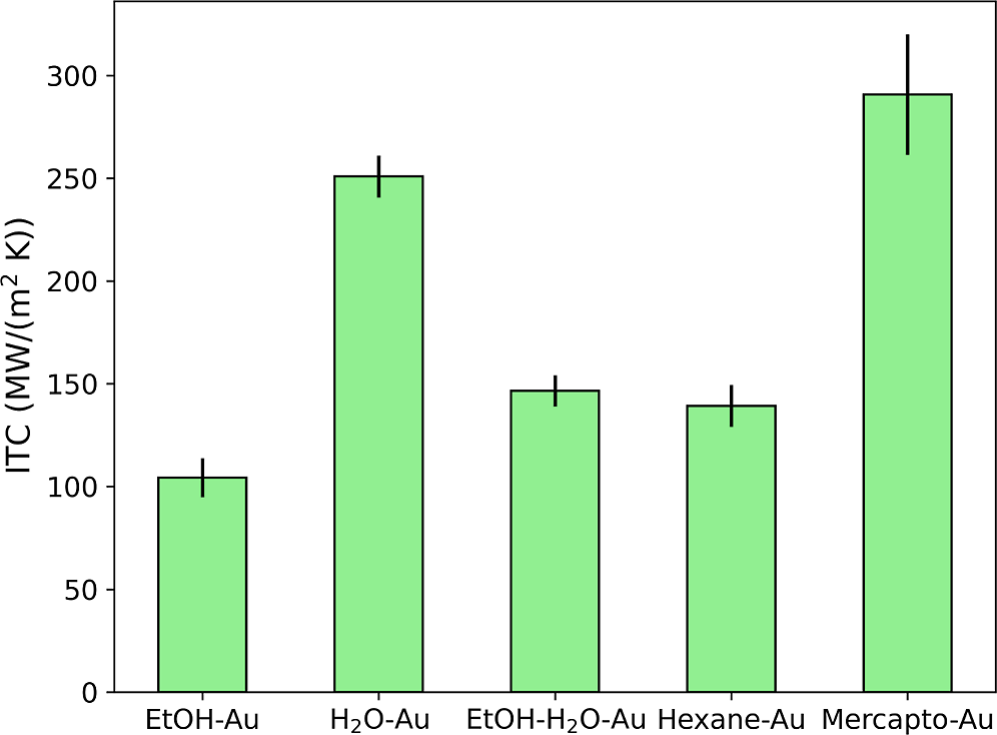
Interfacial thermal conductance of bare gold surface in contact with ethanol (EtOH–Au), water (H_2_O) or the 50:50 mole fraction ethanol–water mixture (EtOH–H_2_O–Au). We also show results for the gold surfaces functionalized with hydrophobic hexanethiol (Hexane-Au) and hydrophilic mercapto hexanol (Mercapto-Au) ligands.

We have conducted additional structural analyses to identify the interactions between ethanol and water, as well as the ligands, and their correlation with interfacial heat transport. Figure S5 illustrates the radial distribution functions (RDFs) between oxygen and hydrogen in both water and ethanol, as well as the oxygen and hydrogen atoms in the mercaptohexanol monolayer. These RDFs demonstrate that water forms strong hydrogen bonds with the mercaptohexanol ligand, as indicated by the double peaks in the O–H and H–O correlations. In contrast, the interactions are weakened in the case of ethanol, where the H–O correlation displays a clear depletion and a shift to distances >2 Å. This shift indicates a reduction in the hydrogen bond interaction of ethanol with the ligand compared to water. The ability of water to form stronger hydrogen bonds results in an enhancement of the density of water next to the surface. Therefore, we conclude that water plays a major role in defining the path of heat transport between the monolayer and the solvent.

We performed a similar analysis for the hydrophobic, –CH_3_ terminated monolayer (see Figure S6a,b). The RDF and the corresponding potential of mean force show an attractive interaction between the CH_3_ groups in ethanol and the ligand. This interaction favors a closer contact between the monolayer and ethanol (see Figure 8a) and indicates that the ethanol molecules provide the main path for heat transport between the monolayer and the solvent.

In summary, the NEMD simulations indicate that the increase in interfacial conductance of water–ethanol mixtures at hydrophilic interfaces is primarily driven by strong interactions between water and the monolayer, and the formation of hydrogen bonds between water molecules and the terminal groups of the ligands. In contrast, the total interfacial thermal conductance of the hydrophobic monolayer in contact with water and ethanol is comparable to that of a bare gold surface. This finding supports recent experimental observations related to alkanethiol-coated gold surfaces.^22^ The simulations indicate that the observed trends are linked to a preferential adsorption of ethanol molecules at the hydrophobic monolayer.

### Thermal Transport Across Gold Coated with a Heterogeneous Monolayer

We also present results for interfacial thermal transport across gold surfaces coated with a heterogeneous monolayer. This monolayer consists of hexanethiol and ligands with a structure that mimics that of the catalytic units (Lip-P_2_)­PdCl_2_), which are closely related to the (diphosphine)­PdCl_2_ (pre)­catalysts used in Suzuki–Miyaura cross-coupling reactions.^68^ For the solvent, we employed the 50:50 ethanol–water mole fraction mixture, previously used in such catalytic reactions.^69^

In Figure S3, we present a top view of the heterogeneous monolayer, which has a ratio of catalytic units to hexanethiol of 10:284. The in-plane spacing between the catalytic units is sufficiently large to prevent overlap of their bulky head groups and the surrounding gold surface coated with hexanethiol spacers. We employed the NEMD simulations to quantify the temperature changes across the gold surface and to calculate the expected temperature in the catalytic units, particularly in the active regions. The hot and cold thermostats were set to *T*_Hot_ = 330 K and *T*_Cold_ = 280 K (see Supporting Information for further computational details).

Figure 10a illustrates the temperature and number density profiles in the direction normal to the gold surface. The catalytic units are fully submerged in the ethanol–water solvent (see Figure 10a,b). Ethanol exhibits an increased density near the hexanethiol head groups, while water shows an enhanced density in the region corresponding to the catalytic unit headgroup. This enhancement indicates a stronger interaction between water and the bulky aromatic head groups (see Figure 2). The temperature profiles display significant jumps between the hexanethiol and the water–ethanol solvent, suggesting significant thermal resistance. This finding is consistent with the results presented in the previous section regarding pure hexanethiol monolayers (see Figures 8a and 9).

**Figure 10:**
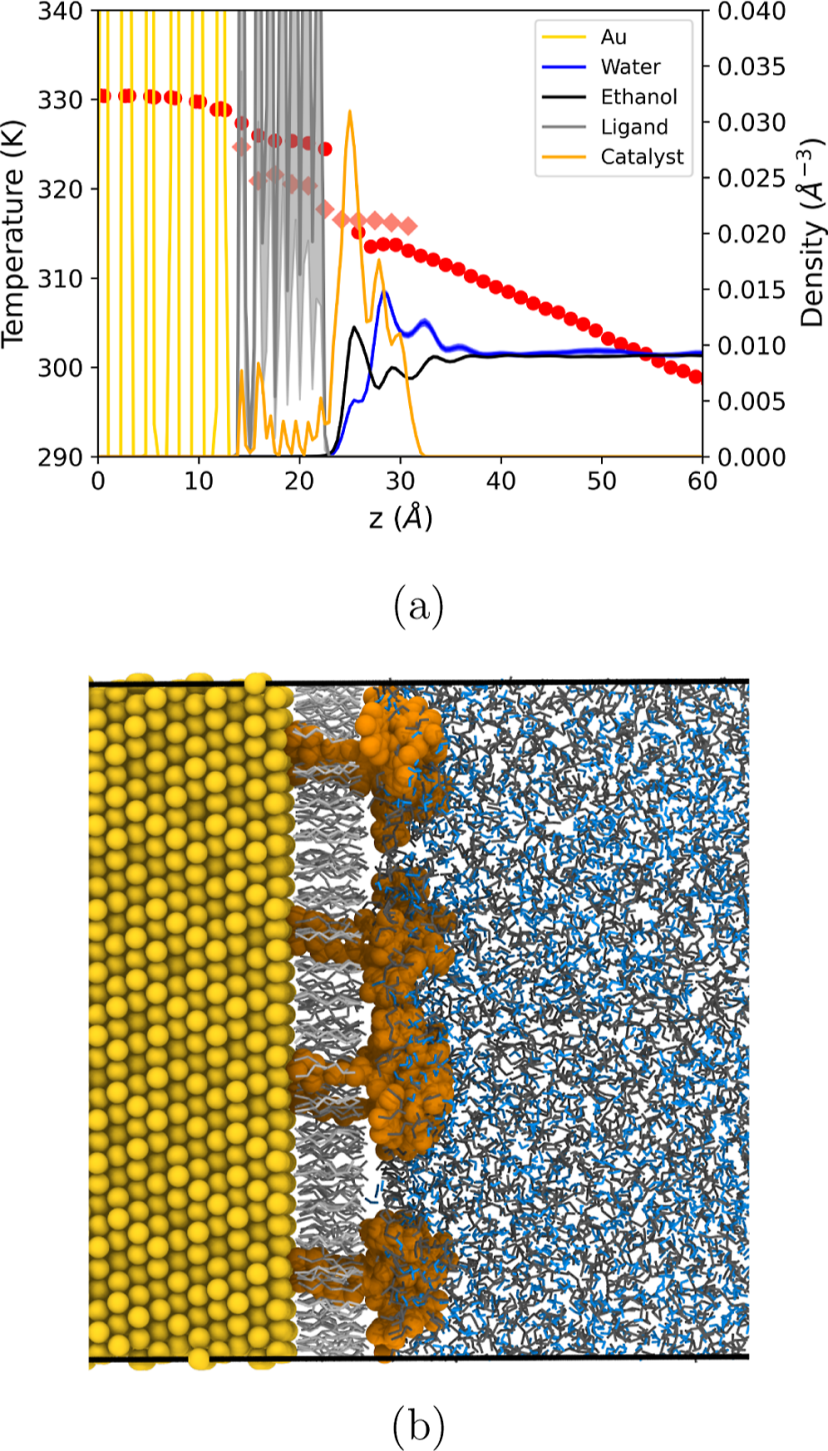
(a) Temperature (circles and diamonds) and number density profiles (full lines) along the heat flux direction. The error bars are of the order of the symbol size and were calculated using three independent replica simulations. (b) A typical configuration showing a lateral view of the heterogeneous monolayer. From left to right: gold (spheres), hexanethiol (shown in bond representation), the catalyst (represented by large spheres), and the ethanol (dark gray-bonds) and water (blue-bonds) solution. The red points representing the temperature profile in the monolayer region correspond to the temperature of the hexanethiol ligands, and in the region above the hexanethiol, following the large temperature jump, to the temperature of the ethanol/water solvent. The diamonds represent the temperature of the catalytic unit.

We have also computed the temperature at the catalytic unit. The temperature profiles reveal differences with respect to the temperature of the hexanethiol ligands, indicating different interfacial thermal transport properties of the two types of ligands. At steady state, the headgroup within the water–ethanol solvent exhibits a higher temperature than the surrounding solvent. This suggests that thermoplasmonic heat transport can maintain the catalytically active sites at a higher temperature than the surrounding environment. This would be beneficial for enhancing bond-forming and bond-breaking processes at the metal center during catalysis, and likely more efficient than external heating of the bulk solution.

We computed the radial distribution functions between the carbon atoms in the aromatic rings of the catalytic unit (A and B rings in Figure 2) and the oxygen atoms in water and ethanol (see Figure S7a,b). Our calculations indicate a significant preference for the catalytic head groups to interact with the water solvent, with an effective attractive interaction that spans ∼7 Å. This attractive interaction is consistent with the enhancement of the water density around the headgroup of the catalytic unit (see Figure 10a). Therefore, the water–ligand interaction provides the key pathway for heat transport between the catalytic unit and the solvent.

Our simulations provide evidence for heterogeneous thermal transport in the monolayer plane. This is expected, as the catalytic units and hexanethiol ligands interact differently with the solvent (refer to the density profiles in Figure 10a). Moreover, the head groups in the catalytic units have a large surface area in contact with the solvent, which may enhance thermal transport through this ligand. To quantify the heterogeneous thermal transport in the monolayer, we analyzed the individual heat rates of the two types of ligands. We introduced this type of analysis in our previous study of Janus nanoparticles.^3^ The total heat rate flowing across one surface, denoted as ${\mathrm{Q̇}}_{T}$ , is divided into two contributions.

8 $$\dot{Q_{T}}={\mathrm{Q̇}}_{c}+{\mathrm{Q̇}}_{h}$$

where $\dot{Q_{c}}$ and $\dot{Q_{h}}$ are the heat rates of the catalytic unit and the hexanethiol hydrophobic ligands, respectively. The total heat flux obtained from the cumulative energy exchanged at the hot and cold thermostats is, Q̇=0.098±0.001⁡μW . The heat rate for hexanethiol is defined as, $\dot{Q_{h}}=G_{K,h}A_{h}\Delta T$ , where *G*_K,h_ = 139.3 ± 10.2 MW/(Km^2^) (see previous section), *A*_h_ is the area occupied by each hexanethiol ligand, which is taken as 17.6 Å^2^/ligand (see the Supporting Information), and Δ*T* = 9.2 K the interfacial temperature jump obtained from the data in Figure 10. Considering 284 hexanethiol ligands, we calculated ${\mathrm{Q̇}}_{h}=0.064\mu W$ and ${\mathrm{Q̇}}_{c}=0.034\mu W$ , which accounts for ∼35% of the total heat rate. This is a significant heat rate, especially given that the hexanethiol ligands occupy about 89% of the gold surface. The enhanced heat rate through the catalytic units is connected to a larger area of the catalytic head groups in contact with the solvent, as well as the stronger interactions between the headgroup and the solvent due to the presence of phosphorus, oxygen, and nitrogen atoms, which contribute to the increased polarity of the head groups. These specific interactions should enhance the efficient and controlled heating of catalytic transformations at the immobilized metal center.

## Conclusion

In this study, we investigated interfacial thermal transport across bare gold surfaces and gold surfaces functionalized with hydrophobic and hydrophilic ligands, in contact with solvents that contain binary mixtures of water and ethanol. These solvents are of interest in a variety of catalytic reactions, such as Suzuki–Miyaura couplings, and provide a greener, nontoxic reaction medium. Using nonequilibrium molecular dynamics simulations and atomistic models, we resolved the interfacial structure and quantified the interfacial thermal conductance.

Using binary solvents introduces new challenges to interfacial thermal transport due to thermodiffusion and the tendency of one of the solvent components to accumulate in either hot (thermophilic) or cold (thermophobic) regions. The models employed in this study accurately reproduce the dependence of density and thermal conductivity on ethanol content. Furthermore, our simulation results for the thermodiffusion of bulk water–ethanol mixtures show excellent agreement with available experimental data. The simulations of the bulk solvent indicate a preference for water to accumulate in the cold region (positive Soret coefficient) for a 50:50 mole fraction. Although we did not examine the typical reactants involved in catalytic reactions in this work, it is evident that these reactants will also experience thermodiffusion in a thermal field. This phenomenon can lead to mass transport toward or away from the catalytic site. This is a topic of great interest for future studies.

However, the behavior of ethanol–water mixtures in contact with gold surfaces is primarily influenced by interfacial effects. Near the bare gold surface, our predictions show ethanol tends to adsorb on gold, resulting in a reduction of the interfacial thermal conductance compared to pure water in contact with gold. When the gold surface is coated with hydrophobic ligands (such as hexanethiol), the contact layer is enriched in ethanol, while water becomes the dominant component of the interfacial layer when the surface is coated with hydrophilic OH-terminated ligands (such as mercaptohexanol). This observation highlights the stronger interaction between water and hydrophilic ligands, which can be attributed to water’s greater ability to form hydrogen bonds with the monolayer.

The distinct interactions of the ethanol–water mixture with hydrophilic and hydrophobic surfaces lead to significant changes in interfacial conductance compared to uncoated surfaces, resulting in either a decrease or an increase in conductance for hydrophobic or hydrophilic surfaces, respectively. These findings align with recent plasmonic sensing experiments and provide insights into the interfacial structure of the solvent, helping to explain the reported experimental behavior.

We conducted an analysis of thermal transport across heterogeneous monolayers containing hydrophobic ligands as spacers, and catalytic units that mimic the structure of the catalysts used in Suzuki–Miyaura coupling reactions (Lip-P_2_)­PdCl_2_. We found a significant difference in the heat flow through the spacer and catalytic units. Despite most of the surface occupied by the spacer ligand (89%), over 35% of the heat is transferred through the ligands of the catalytic units. Furthermore, we observe notable differences in the temperatures of the hydrophobic ligands and catalytic units, which highlight the distinct thermal transport behaviors associated with ligands of complex chemical compositions.

We explain our observations by considering the substantial surface area of the catalytic head groups in contact with the solvent, as well as the stronger interactions between the head groups and the solvent due to the presence of polar groups in the catalytic units. In addition, we show that under stationary conditions, it is possible to maintain a temperature difference of a few degrees between the headgroup of the catalytic unit and the surrounding solvent. Hence, our simulations confirm the potential to achieve significant temperature differences on the nanometer scale,^4^ with heat flows on the order of 0.1 μW, which can be realized in thermoplasmonic experiments involving metal nanomaterials.

Interfacial curvature and surface roughness are common in nanoscale materials. It has been demonstrated that these factors contribute to an increase in interfacial thermal conductance.^29,56^ Their impact on the nanoscale heat transport of catalytic nanomaterials requires further investigation.

Our work demonstrates that the polar binary solvents used in synthetic applications and the combination of hydrophilic and hydrophobic functional groups provide an additional route to control interfacial thermal transport and local temperatures at the nanoscale. We anticipate that the complex heat transport and heterogeneous temperature distributions associated with a multicomponent monolayer consisting of hydrophobic and catalytic units may be interesting for applications in thermoplasmonics, particularly in photoswitchable catalysis. This approach would allow the catalytic activity to be turned on and off through adjusting the irradiation and provide heating at the immobilized catalytic unit, rather than to the bulk solution. Potential benefits include greater control over the catalytic reaction, enhanced efficiency of heating and the ability to conduct sequential reactions, for example, to create block copolymers using a series of different monomers.

## Supplementary Material



## Notes

Detailed simulation protocols, additional thermophysical data, interfacial thermal conductance results, structural analyses, and complete force field parameters are provided in the Supporting Information (PDF)
