# Supplementary material for: Interfacial Thermal Transport of Gold Surfaces Coated with Heterogeneous Monolayers in a Binary Solvent
Source: ACS Appl Mater Interfaces. 2025 Sep 20;17(39):55564–75. doi: 10.1021/acsami.5c12225 (PMC12492324; doi:10.1021/acsami.5c12225)
Supplement: Supplementary file 1 [file am5c12225_si_001.pdf]

Supporting Information: Interfacial thermal transport of gold surfaces coated with heterogeneous monolayers in a binary solvent

Angad Deshmukh,[†] Chao Zeng,[†] James D.E.T. Wilton-Ely,[*,†] and Fernando Bresme[*,†,‡]

†*Department of Chemistry, Molecular Sciences Research Hub Imperial College, W12 0BZ, London, United Kingdom*

‡*Thomas Young Centre for Theory and Simulation of Materials, Imperial College London, London SW7 2AZ, United Kingdom*

E-mail: j.wilton-ely@imperial.ac.uk; f.bresme@imperial.ac.uk

1 General simulation protocol

We employed the following general protocol:

1. The system under study was first equilibrated at a constant temperature and pressure value in the NpT (isobaric-isothermal) ensemble.

2. To generate the heat flux, thermostats were set up in specific regions at both the center and edges of the simulation box. See Figure 1 in the main text.

3. During the production stage, we calculated local properties by dividing the box into bins along the direction of the heat flux. All NEMD simulations were conducted at a constant volume.

2 Simulations of ethanol-water mixtures

Ethanol-water mixtures were simulated to investigate thermodiffusion. Table S1 shows the following mole fraction of ethanol, x_{EtOH}, and the corresponding weight fraction of water, w_{H_2O}.

Table S1: Details of the simulation for ethanol-water mixtures. N_{TOT} represents the total number of molecules used in the simulations at 1 bar of pressure and 300 K within the NPT ensemble.

x_{EtOH}	w_{H_2O}	N_{TOT}
0.05	0.881	840
0.10	0.779	880
0.142	0.703	1000
0.25	0.54	800
0.5	0.281	800
0.75	0.115	800

1. Initialization and Equilibration: Ethanol and water molecules were randomly added to the simulation box to achieve the desired mixture composition. The systems were

then equilibrated at a pressure of 1 bar and a temperature of 300 K in the NpT (Nosé-Hoover thermostat and barostat) ensemble for 10^7 timesteps, using an integration timestep of $\delta t = 0.5$ fs. The equations of motion were integrated using the velocity-Verlet algorithm, with a coupling constant of 100 fs for the thermostat and 500 fs for the barostat. The long-range cutoff for dispersion interactions was set to 14 Å, with analytical tail corrections applied for constant pressure simulations.

2. Non-equilibrium simulations: Two sets of thermostats—one cold and two hot—were arranged perpendicular to the z-axis. The cold thermostat, with a width of 5 Å, was positioned in the middle of the simulation box, while the two hot thermostats, each 2.5 Å width, were located at the edges (refer to Figure 1 in the main paper). The length along the z-direction was twice the length of the box in the x and y directions.

   We employed the CSVR (constant temperature velocity rescaling)[S1] thermostats to maintain the temperatures of the thermostat regions. Separate thermostats were used for the ethanol and water molecules within both the hot and cold regions. The total energy exchanged in this case is the sum of the cumulative energy for both ethanol and water.

   The hot regions were maintained at a temperature of 350 K, while the cold thermostat was set at 250 K to create a heat flux along the z-axis. The simulation was conducted for 4×10^6 timesteps to establish the stationary state (constant flux).

3. Production Run and Data Sampling: As shown in Figure 1 in the main paper, our simulation setup generates two opposing heat fluxes between each pair of thermostats. The system was divided into 100 subvolumes (or bins) of equal width, with the plane oriented perpendicular to the direction of heat flux. The properties of the system were calculated for each chunk to create spatial profiles, which were used to analyze thermal and mass transport properties. The production run was conducted over 2.1×10^8 timesteps, with an integration timestep of 0.5 fs, resulting in a total time of 105 ns.

The temperature in each bin, located at position z, was calculated from the atomic kinetic energies of the atoms within the bin using the equipartition theorem:

$$T(z) = \sum_{i=1}^{N \in z} \frac{m_i v_i^2}{k_B N_f} \tag{1}$$

where, m_i and v_i are the atomic mass and velocity of atom i, respectively, k_B is the Boltzmann constant and N_f is the atomic degrees of freedom. For ethanol, which is modelled as a fully flexible molecule, we use $N_f = 3$. For the rigid water molecule, we calculate the temperatures of the oxygen and hydrogen atoms using the appropriate degrees of freedom, applying the equation derived in reference.[S2]

The heat flux was calculated by using the continuity equation:

$$J_q = \frac{\dot{Q}}{2A} \tag{2}$$

where $\dot{Q}$ represents the total heat rate and A is the cross-sectional area of the simulation box. The calculated flux is divided by two to account for generating two opposing heat fluxes, as illustrated in Figure 1 of the main text.

3 Simulations of uncoated gold surfaces

We simulated a gold (111) surface in contact with a 50:50 mole fraction ethanol-water mixture to quantify the interfacial thermal conductance and interfacial structure.

3.1 Simulation setup and equilibration

A gold slab was created in the center of the simulation box by cutting an FCC (111) bulk solid with a lattice constant of $a = 4.078$ Å.[S3] The (111) plane was oriented perpendicular to the z-axis within the simulation box and consisted of 12, 16, and 11 atomic layers along the x, y, and z directions, respectively. The Au atoms interacted through a 12-6 Lennard-Jones

(LJ) potential using an optimized force field.[S3] The dimensions of the simulation box were set to $34.619 \times 39.975 \times 129.994$ Å^3. An equal number of ethanol and water molecules were added on either side of the gold slab, resulting in a total of 2330 solvent molecules. The solvent molecules interacted with gold using a combination of 12-6 LJ potential, applying combination rules: arithmetic for distance and geometric for interaction strength.

The system was simulated at a constant temperature of 300 K and a pressure of 1 bar, using the NPT ensemble with an integration timestep of $\delta t = 0.5$ fs. The coupling constants for the Nosé-Hoover thermostat and barostat were set to 100 fs and 500 fs, respectively. The long-range Lennard-Jones cutoff for the gold atoms was set to 14 Å, and the long-range interactions for the solvent were computed using the Particle-Particle Particle-Mesh (PPPM) method.[S4] The simulations were run for 10^6 timesteps.

Non-equilibrium simulations were conducted using the CSVR thermostat[S1] to maintain a temperature gradient and a heat flux within the system. The gold atoms were thermostatted at a temperature of 350 K to replicate the plasmonic heating of gold nanoparticles. Separate cold thermostats were set at 300 K for the ethanol and water molecules in the regions located at the edges of the simulation box (see Figure 1 in the main paper). The width of the gold slab along the z-axis was 24.982 Å, and the widths of the cold thermostat regions were half the width of the gold slab. The simulation was run for 4×10^6 timesteps until the stationary state was reached.

During the production phase, data were computed using trajectories sampled over 8 ns. The simulation box was divided into bins of width $\frac{a}{\sqrt{3}}$, where $a = 4.078$ Å is the lattice constant of gold.

4 Simulations of functionalized gold surfaces: hydrophobic and hydrophilic alkanethiol ligands

The Au (111) surface was coated with monolayers of 1-hexanethiol (hydrophobic) and 6-mercapto-1-hexanol (hydrophilic) ligands (see Table S2). To quantify thermal transport across the functionalized gold-solvent interface, we calculated the temperature profile of the systems and the corresponding interfacial thermal conductances (ITCs).

Table S2: Types of ligands used in SAM functionalized Au-ethanol-water simulations.

Ligand	Molecular Structure	MD Structure
Hexanethiol	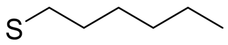	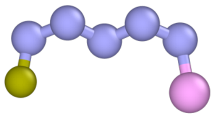
Mercaptohexanol	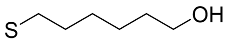	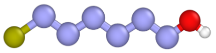

4.1 Simulation setup and equilibration

The Au-solvent setup is similar to what was discussed in the previous section. The Au slab was coated with alkanethiol monolayers on both faces, oriented perpendicular to the z-axis. The monolayers were arranged in a $\sqrt{3} \times \sqrt{3}$ lattice, forming a densely packed layer, in accordance with experimental studies on the binding sites of alkanethiols on the Au surface.[S5] This arrangement corresponds to a c(4x2) superlattice that achieves 100% coverage on the FCC (111) Au surface (see Figure S1).[S6]

Previous simulations of alkanethiol self-assembled monolayers (SAMs) on Au indicated ligand densities of approximately 20 Å^2/chain based on this lattice structure.[S7,S8] In our simulation, the ligand density is approximately 17.5 Å^2/chain, with 80 ligands per face across a cross-sectional area of 1383.955 Å^2. Snapshots of representative configurations are shown in the main text.

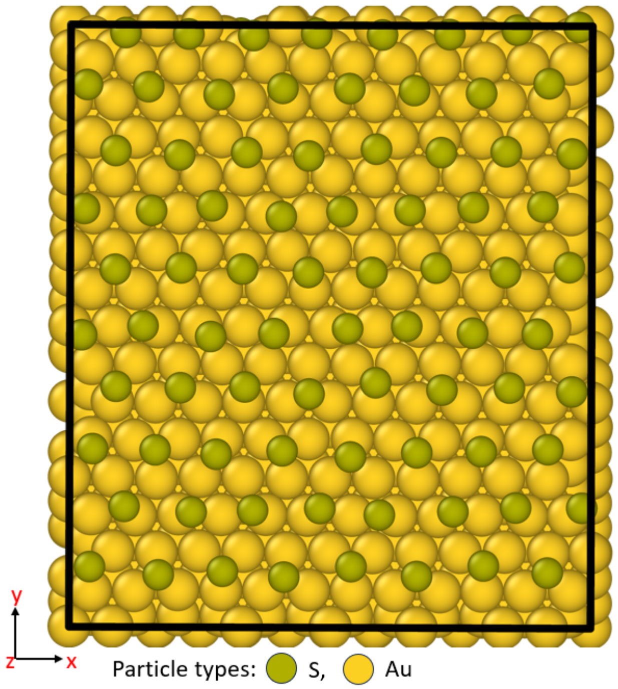

Figure S1: The arrangement of sulfur atoms for a c(4x2) superlattice, which corresponds to 100% surface coverage.

We used Moltemplate[S9] to construct the Self-Assembled Monolayer (SAM) configurations. The length of the simulation box in the z direction was set to values ranging from 165 to 175 Å, depending on the type of ligand used. We incorporated around 3200 solvent molecules in a 50:50 mole ratio of ethanol to water.

The hexanethiol and mercaptohexanol ligands were modeled using a united-atom representation and the TraPPE force field (see Table S2). The ligands interacted with solvent molecules via a Lennard-Jones (LJ) potential of the form 12-6, with the interaction parameters calculated using the Lorentz-Berthelot combination rules. The sulfur atoms in the ligands interacted with gold atoms through an 8-4 nm potential, which was parameterized according to their adsorption energies.[S10]

The systems were equilibrated at a constant temperature of 300 K and a pressure of 1 bar in the NPT ensemble. The coupling constants for the Nosé-Hoover thermostat and barostat were set to 100 fs and 500 fs, respectively. The simulation was performed for 10^6 timesteps, with an integration timestep of $\delta t = 0.5$ fs. The cutoff for the LJ interactions was set 14 Å,

and the electrostatic interactions were computed using the PPPM methid.[S4]

4.2 Non-equilibrium simulations

The setup for the non-equilibrium simulations was similar to that described previously for the uncoated gold slab. A hot thermostat was set at 330 K within the gold (Au) slab, while separate cold thermostats were applied to the ethanol and water molecules located at the edges of the simulation box, with temperatures set at 280 K. The width of each cold region is half the width of the Au slab, which generates a heat flux on either side of the slab. The simulation was conducted for 4×10^6 timesteps with an integration timestep of $\delta t = 0.5$ fs. This simulation time ensured a stable generation of heat flux within the system. The production phase was carried out for 1.6×10^7 timesteps, also with an integration timestep of $\delta t = 0.5$ fs, corresponding to an overall production time of 8 ns.

Since the NEMD simulations are performed at constant volume, the pressure can feature some variations with respect ot the target pressure (see Table S4). Previous studies showed that changes in pressure of a few hundred bars have little imnpact on the thermal transport properties such as thermodiffusion (see e.g.[S11]).

The tytpical heat fluxes for gold functionalised surfaces were $1.99 \pm 0.02 \times 10^9$, $2.73 \pm 0.01 \times 10^9$, $1.77 \pm 0.02 \times 10^9$ W/m^2 for hexane, mercapto and hexane+catalyst functionalised surfaces. Figure S2 shows an example of the cumulative energy for a gold surface coated with mercaptohexanol ligands, illustrating the excellent energy conservation of our method.

4.3 Simulations of functionalized gold surfaces: heterogeneous catalytic layers

The Au slab was functionalized with ligands mimicking molecular structures relevant to catalytic applications.[S12] The catalytic ligand, features a lipoic acid backbone and a phenylphos-

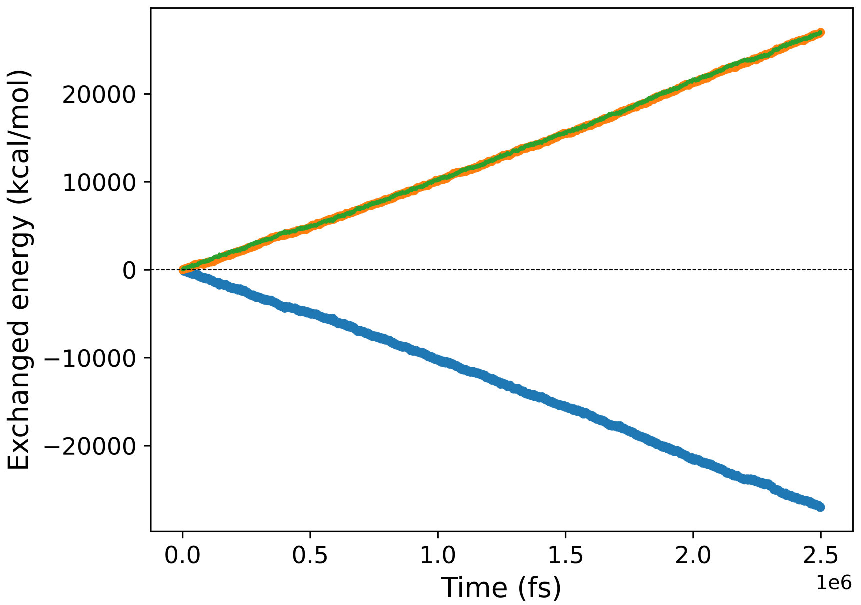

Figure S2: Energy exchanged in the cold (positive values) and hot (negative values) thermostats. We have superimposed the accumulated energy of the cold thermostat with the energy of the hot thermostat. The overlap between both lines demonstrates excellent energy conservation. The results correspond to a simulation of a gold surface coated with mercaptohexanol ligands.

phine head group (see Figure 2 in the main text). It binds covalently to the Au surface through disulfide linkages. The valency of phosphorus in the ligand is maintained at 3, resulting in an overall charge of 0. The catalytic ligand was modeled using the Automated Topology Builder (ATB),[S13] which generated interaction parameters based on the GROMOS 54A7 force field .[S14] ATB was employed to conduct automated quantum mechanical calculations to obtain these parameters. Subsequently, Moltemplate[S9] processed the ATB topology files for our LAMMPS molecular dynamics simulations. The carbon atoms in the para position of the top aromatic rings labelled "B" in Figure 2 in the main paper were modeled as united atoms to enable stable simulations. We found that the force field generated with explicit hydrogen in those positions resulted in unstable simulations. The disulfides in the ligand interacted with gold using the 8-4 nm potential, applying the same parameters as those for $S_{alkanethiols}$ - Au.[S10]

Ten catalytic ligands were positioned on either face of the gold (Au-111) surface. The

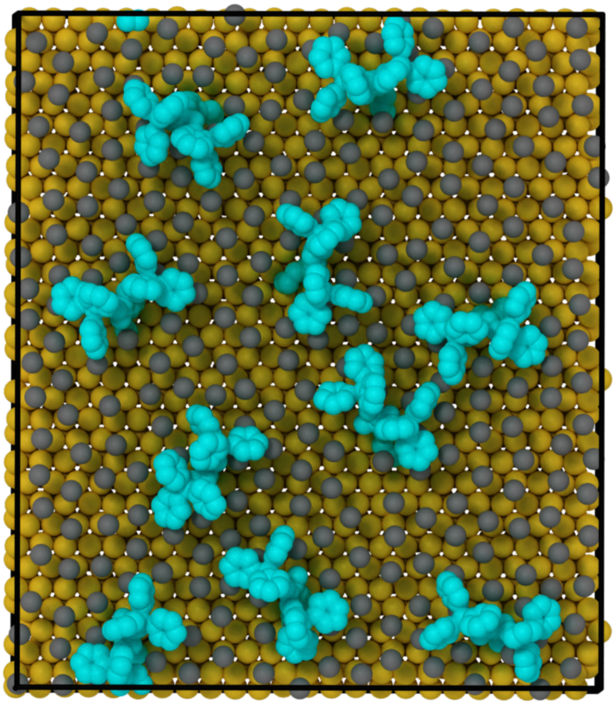

Figure S3: Top view of the heterogeneous monolayers depicting the catalytic ligands (turquoise) and the terminal group (black spheres) of the hexanethiol ligands on the gold surface (orange). Snapshot generated using OVITO.[S15]

surface had a cross-sectional area of 5535.822 $Å^2$ (see Figure S3). To prevent repulsions between the bulky head groups of the catalytic ligands and to avoid direct contact between Au atoms and the solvent, hexanethiol ligands were introduced as spacer molecules. These hexanethiol ligands formed a c(4x2) superlattice structure (see Figure S3).

The total volume of the simulation box was $70 \times 80 \times 206$ $Å^3$, containing 16000 solvent molecules in a 50:50 ethanol-to-water mole ratio. The system was simulated in the NpT ensemble for 10^6 timesteps, maintaining a constant temperature of 300 K and a pressure of 1 bar. An integration timestep of $\delta t = 0.5$ fs was used, along with coupling constants of 100 fs for the Nosé-Hoover thermostat and 2000 fs for the barostat.

The setup and analysis of the NEMD simulations follows the same approach discussed earlier for the homogeneous SAMs. The average z-component of the pressure tensor during the production run was $P_{zz} = 139.191 \pm 6.435\ atm$.

5 Additional thermophysical properties and NEMD results

Table S3: Density of ethanol-water mixtures obtained from NPT simulations at 300 K and 1 bar pressure.

Ethanol mole fraction	Density (g/cm^3)
0.05	0.9783 ± 0.0005
0.1	0.9643 ± 0.0004
0.142	0.9495 ± 0.0004
0.25	0.9137 ± 0.0001
0.5	0.8557 ± 0.0004
0.75	0.8148 ± 0.0002
1.00	0.7811 ± 0.0008

Table S4: The NEMD simulation results for the thermal conductivity, λ (W/(K m)), of ethanol-water mixtures are presented as a function of the ethanol mole fraction (x_{EtOH}). The table includes data for heat flux, $J_q \times 10^{10}$ W/m^2, thermal gradient, $\nabla T \times 10^{10}$ K/m, as well as the average temperature (K) and pressure (atm) of the NEMD runs. Next to each value, we report its respective standard error, which was calculated from the analysis of three independent simulations.

x_{EtOH}	Temperature	Pressure	J_q	∇T	λ
0.05	298.0±0.2	-69.1±5.8	2.82 ± 0.01	4.175 ± 0.006	0.676 ± 0.001
0.10	298.1±0.3	-128±2.9	2.29 ± 0.01	4.103 ± 0.005	0.559 ± 0.001
0.142	297.9±0.4	-33±4.4	1.91 ± 0.0049	3.86 ± 0.01	0.495 ± 0.001
0.25	294.8±0.8	-46±6.0	1.39 ± 0.01	3.932 ± 0.002	0.354 ± 0.001
0.5	293.5±0.9	-68.9±14	0.691 ± 0.001	3.412 ± 0.004	0.2023 ± 0.001
0.75	295.7±0.2	-131±3.4	0.421 ± 0.001	3.012 ± 0.002	0.1398 ± 0.0004

Table S5: Water Soret coefficients $\times 10^{-3}$ K^{-1} calculated from NEMD simulations for different ethanol - water composition at 300 and 320 K. The number in brackets next to the ethanol mole fraction represents the water mass fraction.

	Ethanol mole fraction					
Temperature	0.05(0.881)	0.10(0.779)	0.142(0.703)	0.25(0.54)	0.50(0.281)	0.75(0.115)
300	-5.68 ± 0.45	-4.87 ± 0.34	-3.92 ± 0.21	3.19 ± 0.06	4.81 ± 0.27	2.67 ± 0.10
320	-3.44 ± 0.10	-2.94 ± 0.07	0.65 ± 0.31	0.83 ± 0.59	5.90 ± 0.41	3.80 ± 0.18

Table S6: Interfacial thermal conductance in MW/(K m^2) for various interfaces investigated in this work. EtOH-Au, H_2O-Au, EtOH-H_2O-Au correspond to pure water and the ethanol-water mixture (1:1 composition) in contact with the gold surface. Hexane and mercapto correspond to the hexanethiol and mercaptohexanol-coated gold surfaces in contact with the ethanol-water mixture with 1:1 composition.

Interface	$G_{K,TOTAL}$	$G_{K,Au-ligand}$	$G_{K,ligand}$	$G_{K,ligand-solvent}$
Ethanol-Au	104.3± 9.5	-	-	-
Water-Au	250.9±10.2	-	-	-
Ethanol-Water-Au	146.6± 7.6	-	-	-
Hexane-Au	139.3± 10.2	1193.6±329.2	1180.8±304.7	181.9± 13.9
Mercapto-Au	290.8±29.3	604.0±50.7	893.0±235.1	1507.1±266.6

6 Density and temperature profiles of gold surfaces functionalized with mercapto or hexanethiol ligands

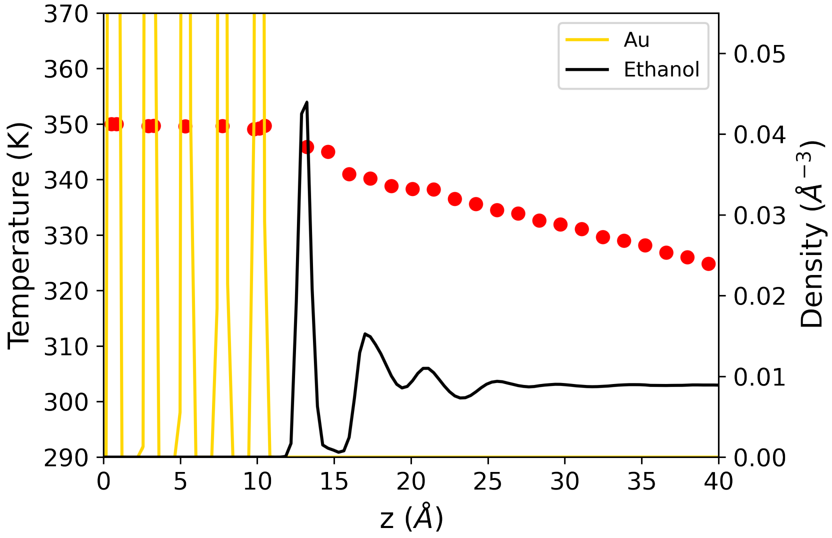

(a)

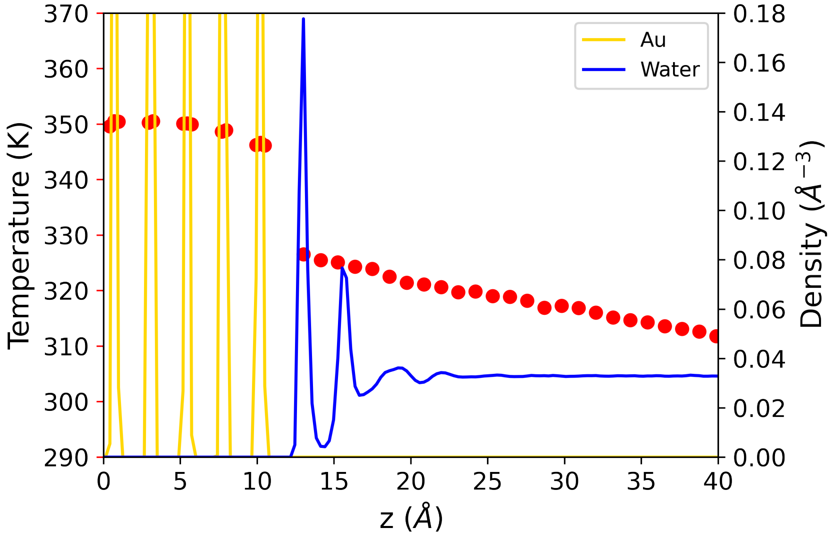

(b)

Figure S4: Temperature (circles) and density (lines) profiles of (a) gold and ethanol and (b) gold and water in the direction normal to the gold surface.

7 Radial distribution functions

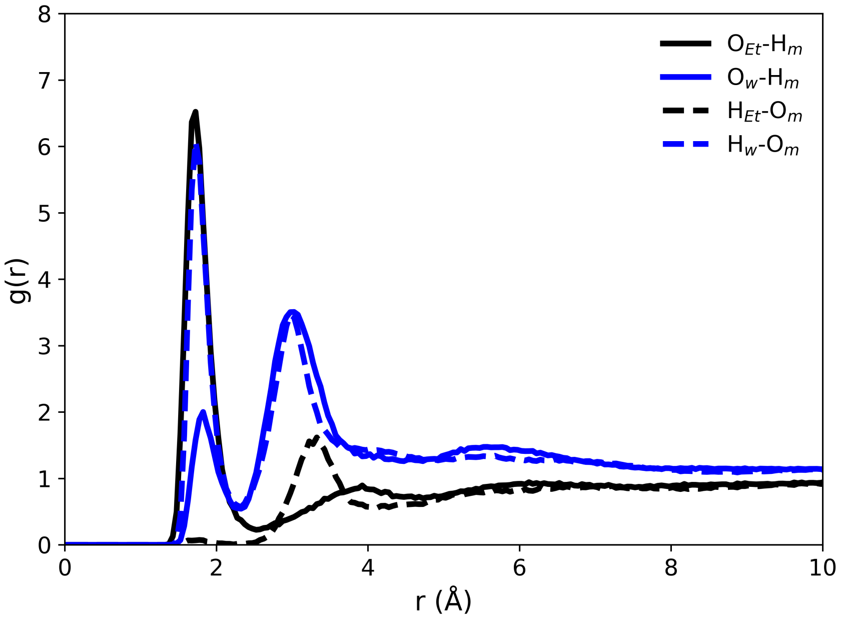

Figure S5: Radial distribution function between the oxygen and hydrogen in ethanol, water and monolayers, indicating the formation of hydrogen bonds between solvent and the gold surface coated with mercaptohexanol ligands. The radial distribution functions are divided by the value at $r = 15$Å.

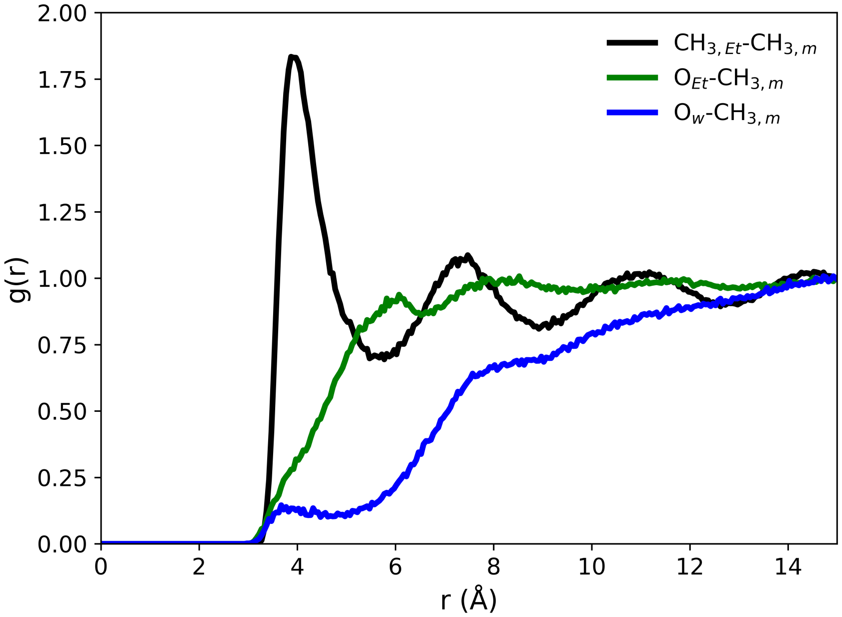

(a)

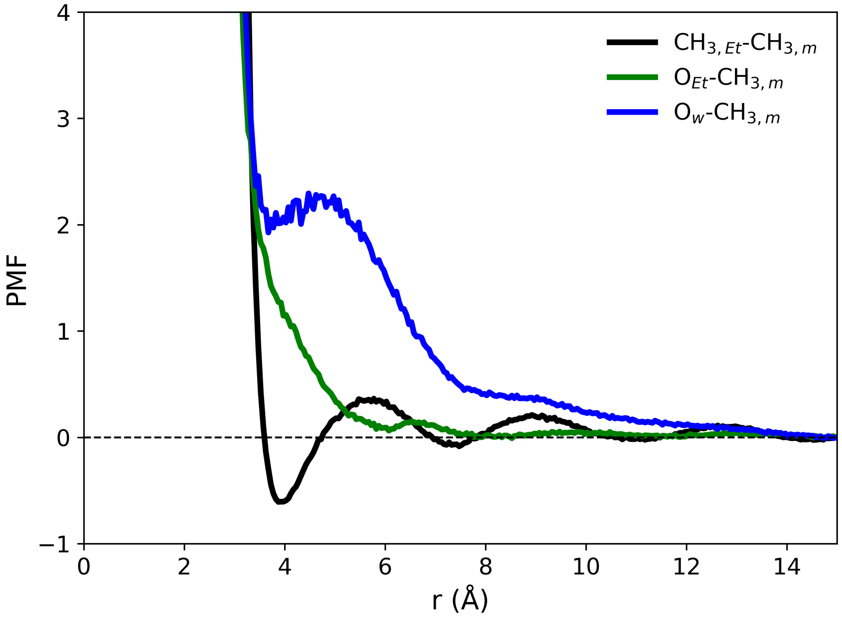

(b)

Figure S6: (a) Radial distribution functions between ethanol and water and the hexanethiol monolayers. The radial distribution functions are divided by the value at $r = 15$Å. (b) Potential of mean force, $\beta PMF(r) = -\log\ g(r)$ ($\beta = 1/k_B T$) for the results shown in panel (a).

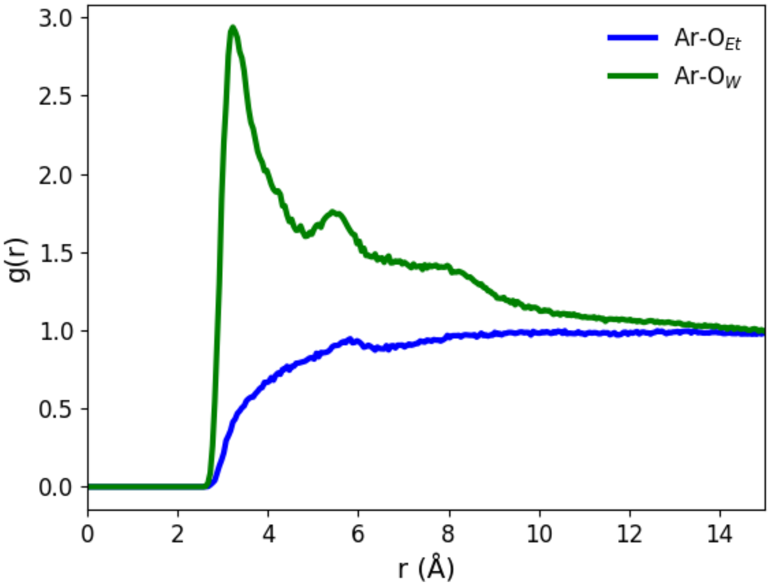

(a)

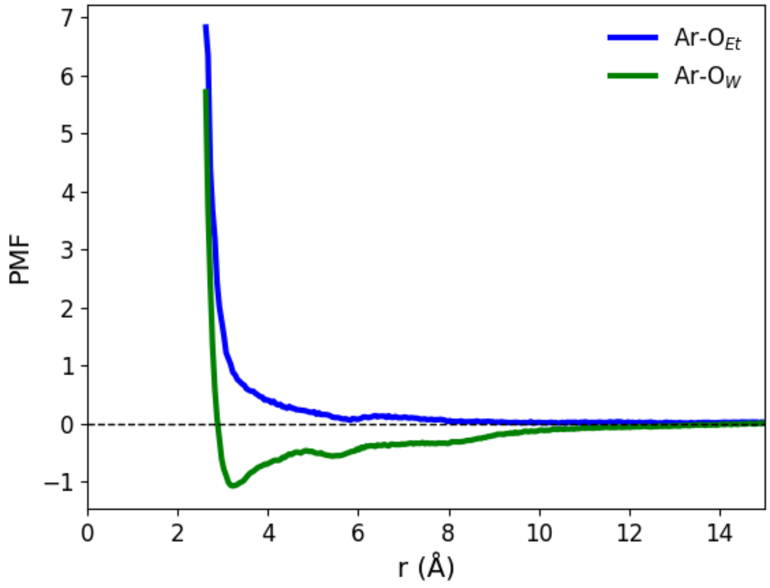

(b)

Figure S7: (a) Radial distribution function between the atoms in the aromatic groups A and B (Ar), as defined in Figure 2 of the main paper, and the oxygen atoms in ethanol (O_{Et}) and water (O_W) molecules. The radial distribution functions are divided by the value at $r = 15$Å. (b) Potential of mean force, $\beta PMF(r) = -\log\ g(r)$ ($\beta = 1/k_BT$) for the results shown in panel (a).

8 Forcefield Parameters

Complete bonded and non-bonded parameters used in this work are provided in Tables S7–S22.

8.1 Water

The water molecules in all the simulation setups were modeled using the TIP4P/2005 model. TIP4P/2005[S16] is a 4-site water model which has an uncharged oxygen Lennard-Jones interaction site while the two hydrogen atoms interact electrostatically. The oxygen charge is located on a dummy site "M", which is on the H-O-H angle bisector at a distance of 0.1546 Åfrom the oxygen atom. The bonds and angles in the molecule are modelled with rigid constraints, and the non-bonded interactions are modelled using the Lennard-Jones potential.

Table S7: Bond parameters for water molecule.

Bonds	r_0 (Å)
O - H	0.9572

Table S8: Angle parameters for water molecule.

Angles	θ_0 (°)
H - O - H	104.52

Table S9: Non-bonded interaction parameters for water molecule.

Interaction	Parameters	
	ϵ (kcal/mol)	σ (Å)
O - O	0.1852	3.1589
H - H	0	0

Table S10: Atom specifications for water molecule.

Particle Types	Mass (g/mol)	Charge (e)
M (O)	15.9994	-1.1128
H	1.00794	0.5564

8.2 Ethanol, hexanethiol and mercaptohexanol

The hydrocarbon molecules, i.e., ethanol (in the solvent mixture), hexanethiol and mercaptohexanol (ligands) are modeled using TraPPE[S17–S19] forcefields as a United-Atom (UA) model. The bonds and angles are modeled using harmonic potential, whereas the dihedrals are modelled using OPLS[S20] potential for ethanol and multi/harmonic 1-4 interaction for the ligands. The non-bonded interactions are modeled using Lennard-Jones potential.

Table S11: Bond parameters for hydrocarbons in the united-atom model.

Bonds	Parameters	
	K_r (kcal/(mol Å^2))	r_0 (Å)
O - H	552.9917	0.945
CH_2 - $CH_{2,3}$	309.9336	1.54
CH_2 - O	320.0803	1.43
S - CH_2	227.01	1.82

Table S12: Angle parameters for hydrocarbons in the united-atom model.

Angles	Parameters	
	K_θ (kcal/(mol rad^2))	θ_0 (°)
S - CH_2 - CH_2	62.1250	114
CH_2 - CH_2 - $CH_{2,3}$	62.1250	114
$CH_{2,3}$ - CH_2 - O	50.076	109.47
CH_2 - O - H	55.044	108.50

Table S13: Dihedral parameters for hydrocarbons in the united-atom model.

Dihedrals	Parameters			
	K_1 (kcal/mol)	K_2 (kcal/mol)	K_3 (kcal/mol)	K_4 (kcal/mol)
CH_3 - CH_2 - O - H (OPLS)	0.4169	-0.0579	0.3734	0
S - CH_2 - CH_2 - CH_2	0.969	1.935	0.131	-3.0348
CH_2 - CH_2 - CH_2 - $CH_{2,3}$	0.969	1.935	0.131	-3.0348
CH_2 - CH_2 - CH_2 - O	0.8048	2.0450	0.1010	-2.9518
CH_2 - CH_2 - O - H	0.3252	3.2677	0.0553	-0.7195

Table S14: Non-bonded interaction parameters for TraPPE forcefield.

Interaction	Parameters	
	ϵ (kcal/mol)	σ (Å)
S - S	0.39701	4.45
CH_2 - CH_2	0.0914	3.95
CH_3 - CH_3 (Ethanol)	0.1947	3.75
CH_3 - CH_3 (Hexanethiol)	0.2265	3.93
O - O	0.1848	3.02
H - H	0	0

Table S15: Atom specifications for the united-atom model.

Particle Types	Mass (g/mol)	Charge (e)
CH_3	15.035	0
CH_2	14.027	0
CH_2 (Ethanol)	14.027	0.265
S	32.06	0
O	15.9994	-0.7
H	1.00794	0.435

8.3 Catalytic Ligand

The forcefields for the catalytic ligands were generated with the ATB (Automated Topology Builder)[S21] server in GROMOS 54A7 forcefield.[S22] The bonds, angles and dihedrals are modeled using the harmonic potential. The non-bonded interactions are modeled using Lennard-Jones potential. Refer to Figure S8 for information on the labels used in the tables below.

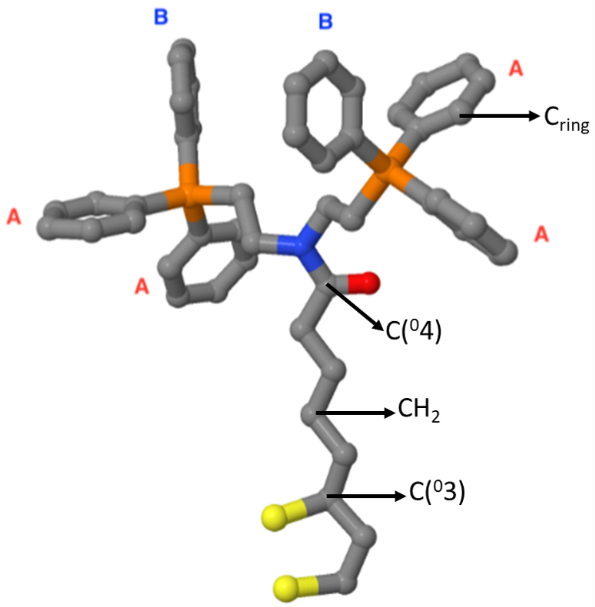

Figure S8: Schematic structure of the catalytic ligand. The spheres are color coded as: yellow = sulfur, grey = carbon, red = oxygen, blue = nitrogen, orange = phosphorus. The labels indicate carbon types with different degrees of substitution.

Table S16: Bond parameters for the catalytic ligand.

Bonds	Parameters	
	K_r (kcal/(mol Å^2))	r_0 (Å)
S - CH_2	227.01	1.82
S - C(°3)	300.044	1.87
CH_2 - CH_2	309.93	1.54
CH_2 - C(°3)	239.006	1.54
C(°3) - H	349.274	1.09
CH_2 - C(°4)	179.254	1.56
C(°4) = O	600.242	1.23
C(°4) - N	399.792	1.43
CH_2 - N	299.976	1.48
CH_2 - P	155.354	1.90
C_{ring} - P	300.044	1.87
C_{ring} - C_{ring}	399.904	1.39
C_{ring} - H_{ring}	349.274	1.09

Table S17: Angle parameters for the catalytic ligand.

Angles	Parameters	
	K_θ (kcal/(mol rad^2))	θ_0 (°)
S - CH_2 - CH_2	55.099	113.0
S - C(°3) - CH_2	55.099	113.0
S - C(°3) - H	52.477	107.57
CH_2 - C(°3) - H	30.173	109.50
CH_2 - CH_2 - CH_2	55.114	111.0
CH_2 - CH_2 - C(°3)	55.114	111.0
CH_2 - CH_2 - C(°4)	55.127	109.5
CH_2 - C(°4) = O	60.072	121.0
CH_2 - C(°4) - N	50.116	120.0
O = C(°4) - N	59.893	124.0
C(°4) - N - CH_2	59.772	116.0
N - CH_2 - CH_2	55.114	111.0
CH_2 - CH_2 - P	50.116	120.0
CH_2 - P - C_{ring}	50.171	108.0
P - C_{ring} - C_{ring}	50.116	120.0
C_{ring} - P - C_{ring}	180.460	109.0
C_{ring} - C_{ring} - C_{ring}	50.116	120.0
C_{ring} - C_{ring} - H_{ring}	45.187	120.0

Table S18: Dihedral parameters for the catalytic ligand.

Dihedrals	Parameters		
	K_1 (kcal/mol)	d (+1 or -1)	n (>/= 0)
S - CH_2 - CH_2 - C(°3)	1.415	+1	3
CH_2 - CH_2 - C(°3) - CH_2	1.415	+1	3
S - C(°3) - CH_2 - CH_2	1.415	+1	3
C(°3) - CH_2 - CH_2 - CH_2	1.415	+1	3
CH_2 - CH_2 - CH_2 - CH_2	1.415	+1	3
CH_2 - CH_2 - CH_2 - C(°4)	1.415	+1	3
CH_2 - CH_2 - C(°4) - N	0.239	+1	6
CH_2 - C(°4) - N - CH_2	8.006	-1	2
N - CH_2 - CH_2 - P	1.415	+1	3
CH_2 - CH_2 - P - C_{ring}	0.239	-1	3
C_{ring} - P - C_{ring} - C_{ring}	0.239	-1	6
CH_2 - P - C_{ring} - C_{ring}	0	+1	1
P - C_{ring} - C_{ring} - C_{ring}	0	+1	1
P - C_{ring} - C_{ring} - H_{ring}	0	+1	1
O = C(°4) - N - CH_2	0	+1	1
C(°4) - N - CH_2 - CH_2	0	+1	1
CH_2 - CH_2 - C(°4) = O	0	+1	1
H - C(°3) - CH_2 - CH_2	0	+1	1
CH_2 - C(°3) - CH_2 - CH_2	0	+1	1
CH_2 - CH_2 - C(°3) - H	0	+1	1
CH_2 - CH_2 - C(°3) - S	0	+1	1

Table S19: Non-bonded interaction parameters for GROMOS 54A7 forcefield.

Interaction	Parameters	
	ϵ (kcal/mol)	σ (Å)
S - S	0.310	3.56
CH_2 - CH_2	0.0981	4.07
C(°3) - C(°3)	0.066	3.58
H - H	0	0
C(°4) - C(°4)	0.245	2.81
O - O	0.119	3.4
N - N	0.22	3.64
P - P	0.585	3.38
C_{ring} - C_{ring}	0.073	3.52
H_{ring} - H_{ring}	0	0

Table S20: Atom specifications for the catalyst molecule.

Particle Types	Mass (g/mol)	Charge (e)
CH_2	14.027	0
C(°3)	12.011	-0.003
C(°4)	12.011	-0.071
C_{ring}	14.027	-0.048
S	32.06	0
P	30.973	0.388
N	14.0067	-0.304
O	15.9994	-0.295
H	1.00794	0.435

8.4 Gold

The gold slab is modeled using Heinz forcefield.[S23] The non-bonded interactions with hexanethiol and solvent (water and ethanol) molecules are described using 12-6 LJ potential. The Au-S covalent bond is modeled using 8-4 nm potential.

Table S21: Non-bonded interaction parameters for gold atoms.

Interaction	Type	Parameters
Au - Au	LJ	ϵ = 5.29 (kcal/mol), σ = 2.629 (Å)
Au - S	nm	E_0 = 9.2256 (kcal/mol), r_0 = 2.9 (Å)

Table S22: Atom specifications for gold atom.

Particle Types	Mass (g/mol)	Charge (e)
Au	196.966	0

All the non-bonded cross-interaction parameters between dissimilar particle types are calculated using Lorentz-Berthelot combining rule.

References

(S1) Bussi, G.; Donadio, D.; Parrinello, M. Canonical sampling through velocity rescaling. *The Journal of Chemical Physics* **2007**, *126*, 014101.

(S2) Olarte-Plata, J. D.; Bresme, F. Thermal conductance of the water–gold interface: The impact of the treatment of surface polarization in non-equilibrium molecular simulations. *The Journal of Chemical Physics* **2022**, *156*.

(S3) Heinz, H.; Vaia, R. A.; Farmer, B. L.; Naik, R. R. Accurate Simulation of Surfaces and Interfaces of Face-Centered Cubic Metals Using 126 and 96 Lennard-Jones Potentials. *The Journal of Physical Chemistry C* **2008**, *112*, 17281–17290.

(S4) Hockney, R. W.; Eastwood, J. W. *Computer Simulation Using Particles*; Adam Hilger: New York, 1989.

(S5) Kondoh, H.; Iwasaki, M.; Shimada, T.; Amemiya, K.; Yokoyama, T.; Ohta, T.; Shimomura, M.; Kono, S. Adsorption of Thiolates to Singly Coordinated Sites on Au(111) Evidenced by Photoelectron Diffraction. *Physical Review Letters* **2003**, *90*, 066102, PRL.

(S6) Poirier, G. E.; Tarlov, M. J. The c(4X2) Superlattice of n-Alkanethiol Monolayers Self-Assembled on Au(111). *Langmuir* **1994**, *10*, 2853–2856, doi: 10.1021/la00021a001.

(S7) Vasumathi, V.; Cordeiro, M. N. D. S. Molecular dynamics study of mixed alkanethiols covering a gold surface at three different arrangements. *Chemical Physics Letters* **2014**, *600*, 79–86.

(S8) Ghorai, P. K.; Glotzer, S. C. Molecular Dynamics Simulation Study of Self-Assembled Monolayers of Alkanethiol Surfactants on Spherical Gold Nanoparticles. *The Journal of Physical Chemistry C* **2007**, *111*, 15857–15862, doi: 10.1021/jp0746289.

(S9) Jewett, A. I.; Stelter, D.; Lambert, J.; Saladi, S. M.; Roscioni, O. M.; Ricci, M.; Autin, L.; Maritan, M.; Bashusqeh, S. M.; Keyes, T.; Dame, R. T.; Shea, J.-E.; Jensen, G. J.; Goodsell, D. S. Moltemplate: A Tool for Coarse-Grained Modeling of Complex Biological Matter and Soft Condensed Matter Physics. *Journal of Molecular Biology* **2021**, *433*, 166841.

(S10) Tay, K. A.; Bresme, F. Wetting Properties of Passivated Metal Nanocrystals at Liquid-Vapor Interfaces: A Computer Simulation Study. *Journal of the American Chemical Society* **2006**, *128*, 14166–14175.

(S11) Di Lecce, S.; Albrecht, T.; Bresme, F. A computational approach to calculate the heat of transport of aqueous solutions. *Scientific Reports* **2017**, *7*, 44833.

(S12) Suzuki, A. Recent advances in the cross-coupling reactions of organoboron derivatives with organic electrophiles, 1995–1998. *Journal of Organometallic Chemistry* **1999**, *576*, 147–168.

(S13) Malde, A. K.; Zuo, L.; Breeze, M.; Stroet, M.; Poger, D.; Nair, P. C.; Oostenbrink, C.; Mark, A. E. An Automated Force Field Topology Builder (ATB) and Repository: Version 1.0. *Journal of Chemical Theory and Computation* **2011**, *7*, 4026–4037, doi: 10.1021/ct200196m.

(S14) Schmid, N.; Eichenberger, A. P.; Choutko, A.; Riniker, S.; Winger, M.; Mark, A. E.; van Gunsteren, W. F. Definition and testing of the GROMOS force-field versions 54A7 and 54B7. *European Biophysics Journal* **2011**, *40*, 843–856.

(S15) Stukowski, A. Visualization and analysis of atomistic simulation data with OVITO–the Open Visualization Tool. *Modelling and Simulation in Materials Science and Engineering* **2010**, *18*, 015012.

(S16) Abascal, J. L. F.; Vega, C. A general purpose model for the condensed phases of water: TIP4P/2005. *The Journal of Chemical Physics* **2005**, *123*, 234505.

(S17) Lubna, N.; Kamath, G.; Potoff, J. J.; Rai, N.; Siepmann, J. I. Transferable Potentials for Phase Equilibria. 8. United-Atom Description for Thiols, Sulfides, Disulfides, and Thiophene. *The Journal of Physical Chemistry B* **2005**, *109*, 24100–24107, doi: 10.1021/jp0549125.

(S18) Chen, B.; Potoff, J. J.; Siepmann, J. I. Monte Carlo Calculations for Alcohols and Their Mixtures with Alkanes. Transferable Potentials for Phase Equilibria. 5. United-Atom Description of Primary, Secondary, and Tertiary Alcohols. *The Journal of Physical Chemistry B* **2001**, *105*, 3093–3104, doi: 10.1021/jp003882x.

(S19) Eggimann, B. L.; Sun, Y.; DeJaco, R. F.; Singh, R.; Ahsan, M.; Josephson, T. R.; Siepmann, J. I. Assessing the Quality of Molecular Simulations for Vapor–Liquid Equilibria: An Analysis of the TraPPE Database. *Journal of Chemical & Engineering Data* **2020**, *65*, 1330–1344.

(S20) Jorgensen, W. L.; Maxwell, D. S.; Tirado-Rives, J. Development and testing of the OPLS all-atom force field on conformational energetics and properties of organic liquids. *Journal of the American Chemical Society* **1996**, *118*, 11225–11236.

(S21) Malde, A. K.; Zuo, L.; Breeze, M.; Stroet, M.; Poger, D.; Nair, P. C.; Oostenbrink, C.; Mark, A. E. An Automated Force Field Topology Builder (ATB) and Repository: Version 1.0. *Journal of Chemical Theory and Computation* **2011**, *7*, 4026–4037, Publisher: American Chemical Society.

(S22) Schmid, N.; Riniker, S.; Eichenberger, A. P.; Choutko, A.; Winger, M.; Mark, A. E.; van Gunsteren, W. F. Definition and testing of the GROMOS force-field versions 54A7 and 54B7. *European Biophysics Journal* **2011**, *40*, 843–856.

(S23) Heinz, H.; Vaia, R. A.; Farmer, B. L.; Naik, R. R. Accurate Simulation of Surfaces and Interfaces of Face-Centered Cubic Metals Using 126 and 96 Lennard-Jones Potentials. *The Journal of Physical Chemistry C* **2008**, *112*, 17281–17290.
